# Supplementary material for: Harmful dimensions of medical culture in relation to physician burnout: A cross-sectional study
Source: PLOS Ment Health. 2025 Apr 17;2(4):e0000301. doi: 10.1371/journal.pmen.0000301 (PMC12798346; doi:10.1371/journal.pmen.0000301)
Supplement: S1 Tables — Table A. Identified potentially harmful professional norms of the medical culture following the review of literature with respective items and references. Items 4 to 10 are items of the SOSS-D (The Stigma of Occupational Stress Scale for Doctors)[29]. Table B. Main parameters of the final EFA solution. This table includes loading parameter estimates, Cronbachs’ alpha (α), Eigenvalues and Variance explained. N = 501. 24 items. Factor loadings in EFA> |0.40| are presented. For EFA; F1 = Work Priority Strain, F2 = Existential Significance of Being a Physician, F3 = Physician’s Moral Obligation to Patients and Colleagues, F4 = Colleagues’ Stigma towards PB, F5 = Personal Stigma towards PB, F6 = Physician’s Discomfort with Patient Role; F7 = Physician’s Central Identity Role, F8 = Sacrificial Nature of Medical Practice. Table C. Communalities of the final EFA solution before and after extraction. Extraction Method: Principal Axis Factoring. Table D. Spearman rho’s correlations (ρ) between the EFA factors. in subsample 1 (N = 501). *The correlation is significant at p<0.01 (1-tailed). **The correlation is significant at p<0.001 (1-tailed). One-tailed correlations were privileged, as we suspected positive correlations between the factors, representing distinct but interdependent dimensions of the same underlying concepts (harmful dimensions of the medical culture). Table E. Description and model fit indices for the different HDMC models. N = 501. χ2 = chi-squared; Df = degrees of freedom; CFI = comparative fit index; TLI = Tucker–Lewis index; RMSEA = root mean square error of approximation; SRMR = standardized root mean square residual. Model 1 is the model that was derived from the EFA. Model 5 is the final retained model. Table F. Standardized structural coefficients (CFA) for the final HDMC Model. Results are presented along with their respected 95% confidence intervals and reliability estimates, estimated in the total sample (N = 1002) and on 21 items. All standardized [file pmen.0000301.s001.docx]

**Table A. Identified potentially harmful professional norms of the medical culture following the review of literature with respective items and references.**

| **Norms** | **Description** | **Items** | **Ref** |
| --- | --- | --- | --- |
| Ethos of invincibility or invulnerability | Being sick belongs to the patient but not physicians. Physicians are invincible and not supposed to get ill. Physicians are not like other humans, they are/should be invulnerable or are expected to be superhumans, impermeable to limitations affecting “normal” humans”. | 1. As a doctor I feel pressure (internally and/or externally) to appear physically and mentally healthy. 2. As a doctor I should not fall ill myself. 3. As a doctor I feel that I have the knowledge and skills to protect myself from disease. | [1–5] |
| Stigma related to professional burnout in physicians  < SOSS-D[6] | Stigma leads to considering burnout and seeking help as a sign of weakness and is a source of professional and social judgement in the medical profession. Stigma is an attitudinal barrier with potentially harmful consequences. Stigma towards burnout is not unique to physicians but seems amplified among them due to their training and medical socialization. In the SOSS-D[6], stigma is measured as a multidimensional phenomenon, including aspects of personal stigma (a person’s stigmatizing attitudes), perceived other stigma (a person’s beliefs about stigmatizing attitudes that other people hold, here colleagues) and perceived structural stigma (policies and practices restricting the opportunities of stigmatized persons). | 1. A doctor who experiences occupational stress or burnout is somehow less capable than a doctor who does not experience those difficulties. 2. Most doctors would consider the experience of occupational stress or burnout as a sign that the doctor is not "right" for the profession. 3. Most doctors would consider a doctor who is experiencing occupational stress or burnout as too sensitive or weak. 4. Where I work, any doctor who is experiencing occupational stress or burnout would be given understanding and support. 5. Where I work, a doctor who is experiencing occupational stress or burnout would probably be better off not telling anyone. 6. I would try to distance myself from a doctor who is experiencing occupational stress or burnout. 7. I would have reservations about working with a doctor who is experiencing difficulties with occupational stress or burnout. | [1,7–12] |
| Perceived incompatibility between the roles of patient and physician | For some physicians, becoming a patient and having to adopt this new identity role when being sick leads to having to give up their other role of physician. It is like if individuals had a “zero-sum identity" and that being a patient or having to go off work for illness was felt as a loss of identity. When physicians are sick, they are confronted to colliding identities. | 1. In the collective imagination, it is the patients, not the doctors, who fall ill. 2. Doctors tend to believe that the role of patient and the role of doctor are incompatible. 3. Falling ill does not call into question my position as a doctor. 4. Having the role of doctor could become a barrier if I should ever have to adopt the role of patient (in case of illness; such as treating myself, taking a few days sick leave, seeing a doctor, etc.). 5. It is not comfortable for a doctor to have to be the patient and take on the role of the sick person. | [1,13–15] |
| Perceived investment and sacrifices to become a physician | To practice medicine, a great dose of effort, investment, and sometimes sacrifice is required. Medicine includes an extensive training and long working hours, requesting harsh commitment from those who which to practice. | 1. I made many sacrifices (long, difficult studies, complicated work, heavy schedules, etc.) to be the doctor I am today. 2. I find that, compared to other professions, practicing medicine requires you to make more sacrifices on a personal level. 3. Becoming a doctor requires a considerable investment, sometimes to the cost of your personal life. | [1,16] |
| Personal and professional life imbalance | The medical culture suggests that work should always come first. Many physicians still suffer from insufficient work-life balance. | 1. My professional life often invades my private life. 2. The demands of my work sometimes prevent me from devoting as much time as I would like to other areas of my life (friends, family, hobbies, etc.). 3. My work often comes before everything else (friends, family, hobbies, me-time). | [12] |
| Profession as identity | Medicine pressures its members to define themselves by their work. Future physicians go through a demanding learning process of socialization that inculcates well-established values into its members. Commitment and identification to the professional role is therefore a central aspect of such a specialized occupation. Identity and career are intertwined in physicians. | 1. My profession as a doctor plays a key role in the way I define myself. 2. In my identity, my role as a doctor takes up much more space than my other roles (parent, brother/sister, friend, neighbor, etc.). 3. My first thought when I have to introduce myself to others is to say that I am a doctor. 4. For me, being a doctor is not just a job, it is who I am more generally. | [17,18] |
| Existential meaning derived from work | Numerous physicians derive a great sense of meaning in life from their profession. For many physicians, medicine is not only a job. It is an identity, a way of being, a life choice, sometimes a vocation or calling. | 1. My work gives my life meaning. 2. My existence would be much less meaningful if I were not a doctor. 3. My work is one of the most important things in my life. | [14] |
| Responsibility towards patients | Physicians experience a deep sense of responsibility, duty and obligation towards patients and their families. These values can lead to sacrificing their own well-being to meet patients’ needs. As stated in the Physicians’ Pledge, when taking their oath, physicians first say “As a member of the medical profession, I solemnly pledge to dedicate my life to the service of humanity, the health and well- being of my patient will be my *first* consideration”. The Hippocratic Oath expresses the dedication of oneself (selflessness) as a part of the person (doctor) who decides to cure for the sick. | 1. I often place my job and my patients before my own well-being. 2. Taking care of my patients is more important than taking care of myself. 3. If I had to temporarily stop working due to illness, I would feel that I was abandoning my patients. 4. I feel very guilty if I am unable to meet my patient’s needs. | [19–21] |
| Responsibility towards colleagues | Physicians are driven by a great sense of duty and obligation towards their fellow physicians. Collegiality is a pillar of medical professionalism. Physicians do not want to let their colleagues down. For example, in case of illness, physicians often seem discouraged to take time off as they know that this will increase the workload of their colleagues, who are often already submerged with work. Solidarity among physicians is important, and sometimes, colleagues’ needs are placed above their own. | 1. I consider it important not to disappoint my fellow doctors. 2. If I were to temporarily stop working due to illness, I would feel guilty about increasing the workload for my colleagues. 3. Team spirit among doctors is central to the way I see my profession. | [22–27] |

**Legend Table A:** Items 4 to 10 are items of the SOSS-D (The Stigma of Occupational Stress Scale for Doctors) [29].

**Table B. Main parameters of the final EFA solution.**

| **Factor →** | **1** | **2** | **3** | **4** | **5** | **6** | **7** | **8** |
| --- | --- | --- | --- | --- | --- | --- | --- | --- |
| **Cronbach’s’ alpha (α) of the factor →** | 0.86 | 0.76 | 0.78 | 0.84 | 0.68 | 0.72 | 0.78 | 0.86 |
| **Eigenvalue (rotated) →** | 5.78 | 2.37 | 1.96 | 1.16 | 0.98 | 0.81 | 0.59 | 0.50 |
| **Total % of variance explained →** | 23.04 | 9.89 | 8.18 | 4.85 | 4.10 | 3.37 | 2.46 | 2.10 |
| **Item ↓ with factor loading** | | | | | | | | |
| My professional life often invades my private life. | 0.79 |  |  |  |  |  |  |  |
| The demands of my work sometimes prevent me from devoting as much time as I would like to other areas of my life (friends, family, hobbies, etc.). | 0.79 |  |  |  |  |  |  |  |
| My work often comes before everything else (friends, family, hobbies, me-time). | 0.78 |  |  |  |  |  |  |  |
| I often place my job and my patients before my own well-being. | 0.63 |  |  |  |  |  |  |  |
| My work gives my life meaning. |  | 0.77 |  |  |  |  |  |  |
| My existence would be much less meaningful if I were not a doctor. |  | 0.68 |  |  |  |  |  |  |
| My work is one of the most important things in my life. |  | 0.67 |  |  |  |  |  |  |
| I made many sacrifices (long, difficult studies, complicated work, heavy schedules, etc.) to be the doctor I am today. |  |  |  |  |  |  |  | -0.71 |
| I find that, compared to other professions, practicing medicine requires you to make more sacrifices on a personal level. |  |  |  |  |  |  |  | -0.86 |
| Becoming a doctor requires a considerable investment, sometimes to the cost of your personal life. |  |  |  |  |  |  |  | -0.81 |
| My profession as a doctor plays a key role in the way I define myself. |  |  |  |  |  |  | -0.52 |  |
| In my identity, my role as a doctor takes up much more space than my other roles (parent, brother/sister, friend, neighbor, etc.). |  |  |  |  |  |  | -0.55 |  |
| My first thought when I have to introduce myself to others is to say that I am a doctor. |  |  |  |  |  |  | -0.59 |  |
| For me, being a doctor is not just a job, it is who I am more generally. |  |  |  |  |  |  | -0.76 |  |
| Having the role of doctor could become a barrier if I should ever have to adopt the role of patient (in case of illness; such as treating myself, taking a few days sick leave, seeing a doctor, etc.). |  |  |  |  |  | -0.68 |  |  |
| It is not comfortable for a doctor to have to be the patient and take on the role of the sick person. |  |  |  |  |  | -0.79 |  |  |
| If I had to temporarily stop working due to illness, I would feel that I was abandoning my patients. |  |  | 0.72 |  |  |  |  |  |
| I feel very guilty if I am unable to meet my patient’s needs. |  |  | 0.79 |  |  |  |  |  |
| I consider it important not to disappoint my fellow doctors. |  |  | 0.54 |  |  |  |  |  |
| If I were to temporarily stop working due to illness, I would feel guilty about increasing the workload for my colleagues. |  |  | 0.56 |  |  |  |  |  |
| Most doctors would consider the experience of occupational stress or burnout as a sign that the doctor is not “right” for the profession. |  |  |  | 0.90 |  |  |  |  |
| Most doctors would consider a doctor who is experiencing occupational stress or burnout as too sensitive or weak. |  |  |  | 0.78 |  |  |  |  |
| I would try to distance myself from a doctor who is experiencing occupational stress or burnout. |  |  |  |  | 0.67 |  |  |  |
| I would have reservations about working with a doctor who is experiencing difficulties with occupational stress or burnout. |  |  |  |  | 0.80 |  |  |  |

**Legend Table B**: This table includes loading parameter estimates, Cronbachs’ alpha (α), Eigenvalues and Variance explained. N = 501. 24 items. Factor loadings in EFA > |0.40| are presented. For EFA; F1 = Work Priority Strain, F2 = Existential Significance of Being a Physician, F3 = Physician’s Moral Obligation to Patients and Colleagues, F4 = Colleagues’ Stigma towards PB, F5 = Personal Stigma towards PB, F6 = Physician’s Discomfort with Patient Role; F7 = Physician’s Central Identity Role, F8 = Sacrificial Nature of Medical Practice.

|  | **Initial** | **Extraction** |
| --- | --- | --- |
| Most doctors would consider the experience of occupational stress or burnout as a sign that the doctor is not "right" for the profession. | 0.56 | 0.82 |
| Most doctors would consider a doctor who is experiencing occupational stress or burnout as too sensitive or weak. | 0.56 | 0.65 |
| I would try to distance myself from a doctor who is experiencing occupational stress or burnout. | 0.30 | 0.45 |
| I would have reservations about working with a doctor who is experiencing difficulties with occupational stress or burnout. | 0.34 | 0.65 |
| Having the role of doctor could become a barrier if I should ever have to adopt the role of patient (in case of illness; such as treating myself, taking a few days sick leave, seeing a doctor, etc.). | 0.39 | 0.53 |
| It is not comfortable for a doctor to have to be the patient and take on the role of the sick person. | 0.39 | 0.63 |
| My professional life often invades my private life. | 0.62 | 0.68 |
| The demands of my work sometimes prevent me from devoting as much time as I would like to other areas of my life (friends, family, hobbies, etc.). | 0.61 | 0.66 |
| My work often comes before everything else (friends, family, hobbies, me-time). | 0.61 | 0.68 |
| I made many sacrifices (long, difficult studies, complicated work, heavy schedules, etc.) to be the doctor I am today. | 0.59 | 0.66 |
| I find that, compared to other professions, practicing medicine requires you to make more sacrifices on a personal level. | 0.55 | 0.67 |
| Becoming a doctor requires a considerable investment, sometimes to the cost of your personal life. | 0.64 | 0.74 |
| My profession as a doctor plays a key role in the way I define myself. | 0.44 | 0.49 |
| In my identity, my role as a doctor takes up much more space than my other roles (parent, brother/sister, friend, neighbor, etc.). | 0.46 | 0.52 |
| My first thought when I have to introduce myself to others is to say that I am a doctor. | 0.39 | 0.55 |
| For me, being a doctor is not just a job, it is who I am more generally. | 0.49 | 0.58 |
| My work gives my life meaning. | 0.41 | 0.56 |
| My existence would be much less meaningful if I were not a doctor. | 0.42 | 0.53 |
| My work is one of the most important things in my life. | 0.46 | 0.55 |
| I often place my job and my patients before my own well-being. | 0.57 | 0.59 |
| If I had to temporarily stop working due to illness, I would feel that I was abandoning my patients. | 0.45 | 0.53 |
| I feel very guilty if I am unable to meet my patient's needs. | 0.50 | 0.62 |
| I consider it important not to disappoint my fellow doctors. | 0.39 | 0.39 |
| If I were to temporarily stop working due to illness, I would feel guilty about increasing the workload for my colleagues. | 0.42 | 0.45 |

**Table C. Communalities of the final EFA solution before and after extraction.**

**Legend Table C:** Extraction Method: Principal Axis Factoring.

**Table D. Spearman rho’s correlations (*ρ)* between the EFA factors.**

|  | **Work Priority Strain** | **Existential Significance of Being a Physician** | **Physician’s Moral Obligation to Patients and Colleagues** | **Colleagues’ Stigma towards PB** | **Personal Stigma towards PB** | **Physician’s Discomfort with Patient Role** | **Physician’s Central Identity Role** | **Self-sacrificing Nature of Medical Practice** |
| --- | --- | --- | --- | --- | --- | --- | --- | --- |
| **Work Priority Strain** | -- |  |  |  |  |  |  |  |
| **Existential Significance of Being a Physician** | 0.10 | -- |  |  |  |  |  |  |
| **Physician’s Moral Obligation to Patients and Colleagues** | 0.37^**^ | 0.24^**^ | -- |  |  |  |  |  |
| **Colleagues’ Stigma towards PB** | 0.22^**^ | 0.03 | 0.30^**^ | -- |  |  |  |  |
| **Personal Stigma towards PB** | 0.15^**^ | 0.15^**^ | 0.13^*^ | 0.13^*^ | -- |  |  |  |
| **Physician’s Discomfort with Patient Role** | 0.36^**^ | 0.46^**^ | 0.27^**^ | 0.09 | 0.19^**^ | -- |  |  |
| **Physician’s Central Identity Role** | 0.37^**^ | -0.01 | 0.21^**^ | 0.31^**^ | 0.08 | 0.03 | -- |  |
| **Self-sacrificing Nature of Medical Practice** | 0.27^**^ | 0.07 | 0.41^**^ | 0.23^**^ | 0.17^**^ | 0.18^*^ | 0.17^**^ | -- |

**Legend Table D**: in subsample 1 (N = 501). *The correlation is significant at p<0.01 (1-tailed). **The correlation is significant at p<0.001 (1-tailed). One-tailed correlations were privileged, as we suspected positive correlations between the factors, representing distinct but interdependent dimensions of the same underlying concepts (harmful dimensions of the medical culture).

**Table E. Description and model fit indices for the different HDMC models.**

| **Measurement model and description** | **# of items** | **χ2** | **Df** | **CFI** | **TLI** | **RMSEA (90% CI)** | **SRMR** |
| --- | --- | --- | --- | --- | --- | --- | --- |
| **1.** First-order model of *eight* distinct but correlated factors extracted from the EFA, representing the concepts of Work Priority Strain; Existential Significance of Being a Physician; Physician’s Moral Obligation to Patients and Colleagues; Colleagues’ Stigma towards PB; Personal Stigma towards PB; Physician’s Discomfort with Patient Role; Physician’s Central Identity Role; and Self-sacrificing Nature of Medical Practice. | 24 | 603.90 | 224 | 0.91 | 0.89 | 0.06 (0.05 to 0.06) | 0.05 |
| **2.** First-order model of *seven* distinct but correlated factors, similar to model 1 but *without* the factor Existential Significance of Being a Physician. | 21 | 454.15 | 168 | 0.93 | 0.91 | 0.06 (0.05 to 0.07) | 0.05 |
| **3.** Second-order one-factor model: the seven latent factors of model 2 measure harmful dimensions of the unique medical culture, therefore loading on one general “harmful dimensions of the medical culture” (HDMC) factor. | 21 | 494.28 | 182 | 0.92 | 0.91 | 0.06 (0.05 to 0.07) | 0.05 |
| **4**. Second-order hierarchical model: the eight factors of model 1 load onto three second-order dimensions (Physician’s Professional Commitment; The Myth of the Invulnerable Physician; and Physician Stigma towards Burnout – *with* the latent factor of Existential Significance of Being a Physician). | 24 | 727.53 | 241 | 0.89 | 0.88 | 0.06 (0.06 to 0.07) | 0.07 |
| 5. Second-order hierarchical model: the seven factors of Model 2 load onto three second-order dimensions (Physician’s Professional Commitment; The Myth of the Invulnerable Physician; and Physician Stigma towards Burnout – *without* the latent factor of Existential Significance of Being a Physician ). | 21 | 469.43 | 179 | 0.92 | 0.91 | 0.06 (0.05 to 0.06) | 0.05 |

**Legend Table E**: N = 501. χ2 = chi-squared; Df = degrees of freedom; CFI = comparative fit index; TLI = Tucker–Lewis index; RMSEA = root mean square error of approximation; SRMR = standardized root mean square residual. Model 1 is the model that was derived from the EFA. Model 5 is the final retained model.

**Table F. Standardized structural coefficients (CFA) for the final HDMC model.**

| **CFA first-order factors →**  **CFA second-order dimensions ↓** | **F1** | **F2** | **F3** | **F4** | **F5** | **F6** | **F7** |
| --- | --- | --- | --- | --- | --- | --- | --- |
| **Physician’s Professional Commitment** (α = 0.84) | 0.83  (0.78 to 0.88) |  |  |  |  | 0.53  (0.46 to 0.59) | 0.82  (0.77 to 0.87) |
| **The Myth of the Invulnerable Physician** (α = 0.75) |  | 0.82  (0.73 to 0.89) |  |  | 0.60  (0.52 to 0.67) |  |  |
| **Physician Stigma towards Burnout** (α = 0.62) |  |  | 0.54  (0.39 to 0.68) | 0.28  (0.17 to 0.39) |  |  |  |

**Legend Table F:** Results are presented along with their respected 95% confidence intervals and reliability estimates, estimated in the total sample (N = 1002) and on 21 items. All standardized coefficients are significant at p-value <0.000. F1 = Work Priority Strain, F2 = Physician’s Moral Obligation to Patients and Colleagues, F3 = Colleagues’ Stigma towards PB, F4 = Personal Stigma towards PB, F5 = Physician’s Discomfort with Patient Role, F6 = Physician’s Central Identity Role, F7 = and Sacrificial Nature of Medical Practice.

**Table G. Standardized covariances of the second-order dimensions for the final HDMC model.**

|  | **Physician’s Professional**  **Commitment** | **The Myth of the Invulnerable Physician** |
| --- | --- | --- |
| **Physician’s Professional Commitment** | -- |  |
| **The Myth of the Invulnerable Physician** | 0.62 (0.54 to 0.79) | -- |
| **Physician Stigma towards Burnout** | 0.56 (0.39 to 0.73) | 0.74 (0.52 to 0.95) |

**Legend Table G:** Results are presented along with their respected 95% confidence intervals (N = 1002).

**Table H. Standardized measurements coefficients (CFA) of each item on the final HDMC model.**

| **First-order factors of CFA Model 5 →**  **Items ↓** | **1** | **2** | **3** | **4** | **5** | **6** | **7** |
| --- | --- | --- | --- | --- | --- | --- | --- |
| 16. My professional life often invades my private life. | 0.80  (0.77 to 0.83) |  |  |  |  |  |  |
| 17. The demands of my work sometimes prevent me from devoting as much time as I would like to other areas of my life (friends, family, hobbies, etc.). | 0.79  (0.76 to 0.82) |  |  |  |  |  |  |
| 18. My work often comes before everything else (friends, family, hobbies, me-time). | 0.78  (0.75 to 0.81) |  |  |  |  |  |  |
| 29. I often place my job and my patients before my own well-being. | 0.72  (0.69 to 0.76) |  |  |  |  |  |  |
| 19. I made many sacrifices (long, difficult studies, complicated work, heavy schedules, etc.) to be the doctor I am today. |  |  |  |  |  |  | 0.79  (0.76 to 0.82) |
| 20. I find that, compared to other professions, practicing medicine requires you to make more sacrifices on a personal level. |  |  |  |  |  |  | 0.79  (0.76 to 0.82) |
| 21. Becoming a doctor requires a considerable investment, sometimes to the cost of your personal life. |  |  |  |  |  |  | 0.85  (0.83 to 0.88) |
| 22. My profession as a doctor plays a key role in the way I define myself. |  |  |  |  |  | 0.73  (0.68 to 0.77) |  |
| 23. In my identity, my role as a doctor takes up much more space than my other roles (parent, brother/sister, friend, neighbor, etc.). |  |  |  |  |  | 0.70  (0.66 to 0.75) |  |
| 24. My first thought when I have to introduce myself to others is to say that I am a doctor. |  |  |  |  |  | 0.63  (0.58 to 0.68) |  |
| 25. For me, being a doctor is not just a job, it is who I am more generally. |  |  |  |  |  | 0.63  (0.58 to 0.68) |  |
| 14. Having the role of doctor could become a barrier if I should ever have to adopt the role of patient (in case of illness; such as treating myself, taking a few days sick leave, seeing a doctor, etc.). |  |  |  |  | 0.74  (0.68 to 0.81) |  |  |
| 15. It is not comfortable for a doctor to have to be the patient and take on the role of the sick person. |  |  |  |  | 0.76  (0.69 to 0.82) |  |  |
| 31. If I had to temporarily stop working due to illness, I would feel that I was abandoning my patients. |  | 0.67  (0.63 to 0.72) |  |  |  |  |  |
| 32. I feel very guilty if I am unable to meet my patient's needs. |  | 0.75  (0.70 to 0.79) |  |  |  |  |  |
| 33. I consider it important not to disappoint my fellow doctors. |  | 0.68  (0.64 to 0.73) |  |  |  |  |  |
| 34. If I were to temporarily stop working due to illness, I would feel guilty about increasing the workload for my colleagues. |  | 0.68  (0.64 to 0.73) |  |  |  |  |  |
| 5. Most doctors would consider the experience of occupational stress or burnout as a sign that the doctor is not "right" for the profession. |  |  | 0.84  (0.78 to 0.90) |  |  |  |  |
| 6. Most doctors would consider a doctor who is experiencing occupational stress or burnout as too sensitive or weak. |  |  | 0.88  (0.82 to 0.95) |  |  |  |  |
| 9. I would try to distance myself from a doctor who is experiencing occupational stress or burnout. |  |  |  | 0.57  (0.44 to 0.72) |  |  |  |
| 10. I would have reservations about working with a doctor who is experiencing difficulties with occupational stress or burnout. |  |  |  | 0.92  (0.71 to 1.14) |  |  |  |

**Legend Table H:** F1 = Work Priority Strain, F2 = Physician’s Moral Obligation to Patients and Colleagues, F3= Colleagues’ Stigma towards PB, F4 = Personal Stigma towards PB, F5 = Physician’s Discomfort with Patient Role; F6 = Physician’s Central Identity Role, F7= Self-sacrificing Nature of Medical Practice (N = 1002).

**Table I. First and second block of the hierarchical multiple regression of the association between the investigated dimensions of medical culture and burnout, while controlling for covariates.**

|  | **Std. B** | **95% CI for Std.B** | ***p*** |
| --- | --- | --- | --- |
| **Block 1. F(7, 963) = 5.047, p < .001, R^2^ = 0.036, R^2^_adjusted_= 0.029** | | | |
| (Constant) |  |  | <0.001 |
| Gender | 0.12 | 0.05 to 0.18 | <0.001 |
| Relationship status | 0.01 | -0.05 to 0.08 | 0.72 |
| Parental status | -0.00 | -0.11 to 0.11 | 0.99 |
| Physician status | 0.03 | -0.05 to 0.11 | 0.48 |
| Specialty | -0.02 | -0.09 to 0.05 | 0.60 |
| Years in practice | -0.7 | -0.17 to 0.2 | 0.11 |
| Hours worked per week | 0.10 | 0.04 to 0.16 | 0.003 |
| **Block 2. F(11, 963) = 36.584, p < .001, R^2^ = 0.297, R^2^_adjusted_= 0.289** | | | |
| (Constant) |  |  | <.001 |
| Gender | .05 | 0.00 to 0.11 | 0.06 |
| Relationship status | -0.01 | -0.07 to 0.05 | 0.71 |
| Parental status | -0.02 | -0.10 to 0.06 | 0.65 |
| Physician status | 0.04 | -0.04 to 0.11 | 0.34 |
| Specialty | -0.06 | -0.12 to 0.00 | 0.04 |
| Years in practice | 0.05 | -0.02 to 0.14 | 0.24 |
| Hours worked per week | -0.03 | -0.11 to 0.05 | 0.39 |
| Existential Significance of Being a Physician | -0.31 | -0.37 to -0.26 | <0.001 |
| Physician’s Professional Commitment | 0.38 | 0.31 to 0.44 | <0.001 |
| The Myth of the Invulnerable Physician | 0.22 | 0.15 to 0.28 | <0.001 |
| Physician Stigma towards Burnout | 0.08 | 0.03 to 0.14 | 0.004 |

**Legend Table I:** Dependent variable is the PB score. The following covariates were controlled in the model in step 1: gender (female referent), relationship status (being in a relationship referent), parental status (having children referent), physician status (resident referent), specialty (specialist referent), years in practice, working hours estimated per week. In step 2, the HDMC and Existential Significance were added. N = 964. Missing values were excluded listwise. Std B = standardized beta. CI = Confidence Interval. By convention, we do not provide standardized betas for the constant of the regression. *Without* the factor Existential Significance of Being a Physician, the three second-order HDMC dimensions significantly contribute to the prediction of burnout, while controlling for the covariates (R^2^ = 0.21).

**Table J. First and second block of the hierarchical multiple regression of the association between the investigated dimensions of medical culture and burnout, while controlling for covariates including social desirability.**

|  | **Std. B** | **95% CI for Std.B** | ***p*** |
| --- | --- | --- | --- |
| **Block 1. F(7, 933) = 4.542, p < 0.001, R^2^ = 0.033, R^2^_adjusted_= 0.026** | | | |
| (Constant) |  |  | <0.001 |
| Gender | 0.11 | 0.04 to 0.18 | 0.001 |
| Relationship status | 0.03 | -0.04 to 0.10 | 0.39 |
| Parental status | -0.01 | -0.10 to 0.08 | 0.82 |
| Physician status | 0.06 | -0,03 to 0.15 | 0.17 |
| Specialty | -0.04 | -0,11 to 0.03 | 0.26 |
| Years in practice | -0.05 | -0,14 to 0.05 | 0.31 |
| Hours worked per week | 0.08 | 0,02 to 0.15 | 0.02 |
| **Block 2. F(11, 933) = 36.493, p < 0.001, R^2^ = 0.315, R^2^_adjusted_= 0.307** | | | |
| (Constant) |  |  | <0.001 |
| Gender | 0.05 | -0,01 to 0.11 | 0.1 |
| Relationship status | 0.00 | -0,05 to 0.06 | 0.86 |
| Parental status | -0.03 | -0,10 to 0.05 | 0.45 |
| Physician status | 0.07 | -0,01 to 0.14 | 0.07 |
| Specialty | -0.08 | -0,14 to -0.02 | 0.008 |
| Years in practice | 0.08 | 0,00 to 0.14 | 0.04 |
| Hours worked per week | -0.04 | -0,11 to 0.02 | 0.18 |
| Existential Significance of Being a Physician | -0.33 | -0,39 to -0.28 | <0.001 |
| Physician’s Professional Commitment | 0.36 | 0,30 to 0.43 | <0.001 |
| The Myth of the Invulnerable Physician | 0.25 | 0,18 to 0.31 | <0.001 |
| Physician Stigma towards Burnout | 0.08 | 0,02 to 0.13 | 0.007 |
| **Block 3. F(12, 933) = 43.992, p < 0.001, R^2^ = 0.364, R^2^_adjusted_= 0.356** | | | |
| (Constant) |  |  | <0.001 |
| Gender | 0.05 | -0,01 to 0.10 | 0.18 |
| Relationship status | 0.01 | -0,04 to 0.07 | 0.64 |
| Parental status | -0.05 | -0,13 to 0.02 | 0.18 |
| Physician status | 0.06 | -0,01 to 0.13 | 0.09 |
| Specialty | -0.06 | -0,11 to 0.00 | 0.04 |
| Years in practice | 0.12 | 0,05 to 0.19 | 0.002 |
| Hours worked per week | -0.03 | -0,10 to 0.05 | 0.40 |
| Existential Significance of Being a Physician | -0.31 | -0,36 to -0.25 | <0.001 |
| Physician’s Professional Commitment | 0.32 | 0,26 to 0.38 | <.001 |
| The Myth of the Invulnerable Physician | 0.19 | 0,13 to 0.36 | <.001 |
| Physician Stigma towards Burnout | 0.07 | 0,01 to 0.12 | .021 |
| Social desirability | -0.24 | -0,30 to -0.19 | <.001 |

**Legend Table J**: Dependent variable is Burnout score. The following covariates were controlled in the model in step 1: gender (female referent), relationship status (being in a relationship referent), parental status (having children referent), physician status (resident referent), specialty (specialist referent), years in practice, working hours estimated per week. In step 2, the HDMC and Existential Significance were added. In step 3, we added social desirability. N = 934. Missing values were excluded listwise. In this regression, 16 cases were excluded as they were considered outliers (> |2| standard residuals) in the model. Std B = standardized beta. CI = Confidence Interval. By convention, we do not provide standardized betas for the regression coefficient. Controlling for social desirability increased the proportion of variance in burnout explained by the model. This was unsurprising, as self-reported burnout may be impacted by the (un)desirability of suffering from burnout, especially so among physicians. Social desirability negatively predicted burnout (Std B = -0.24, *p* < .001), and the three HDMC and Existential Significance remained significant predictors of burnout. Note that results including social desirability need to be interpreted cautiously as the internal consistency of this measure was low in our sample (α = 0.46).

**Table K. Final HDMC measure.**

| *The following statements relate to aspects specific to the medical profession. Please use the scales below to indicate the extent to which you agree or disagree with the following statements.* | | | | | |
| --- | --- | --- | --- | --- | --- |
|  | Totally disagree | Disagree | Neither disagree nor agree | Agree | Totally agree |
| **Physician’s Professional Commitment** |  | | | | |
| 1. My professional life often invades my private life. | ☐ | ☐ | ☐ | ☐ | ☐ |
| 1. The demands of my work sometimes prevent me from devoting as much time as I would like to other areas of my life (friends, family, hobbies, etc.). | ☐ | ☐ | ☐ | ☐ | ☐ |
| 1. My work often comes before everything else (friends, family, hobbies, me-time). | ☐ | ☐ | ☐ | ☐ | ☐ |
| 1. I often place my job and my patients before my own well-being. | ☐ | ☐ | ☐ | ☐ | ☐ |
| 1. I made many sacrifices (long, difficult studies, complicated work, heavy schedules, etc.) to be the doctor I am today. | ☐ | ☐ | ☐ | ☐ | ☐ |
| 1. I find that, compared to other professions, practicing medicine requires you to make more sacrifices on a personal level. | ☐ | ☐ | ☐ | ☐ | ☐ |
| 1. Becoming a doctor requires a considerable investment, sometimes to the cost of your personal life. | ☐ | ☐ | ☐ | ☐ | ☐ |
| 1. My profession as a doctor plays a key role in the way I define myself. | ☐ | ☐ | ☐ | ☐ | ☐ |
| 1. In my identity, my role as a doctor takes up much more space than my other roles (parent, brother/sister, friend, neighbor, etc.). | ☐ | ☐ | ☐ | ☐ | ☐ |
| 1. My first thought when I have to introduce myself to others is to say that I am a doctor. | ☐ | ☐ | ☐ | ☐ | ☐ |
| 1. For me, being a doctor is not just a job, it is who I am more generally. | ☐ | ☐ | ☐ | ☐ | ☐ |
| **The Myth of the Invulnerable Physician** | | | | | |
| 1. If I had to temporarily stop working due to illness, I would feel that I was abandoning my patients. | ☐ | ☐ | ☐ | ☐ | ☐ |
| 1. I feel very guilty if I am unable to meet my patient's needs. | ☐ | ☐ | ☐ | ☐ | ☐ |
| 1. I consider it important not to disappoint my fellow doctors. | ☐ | ☐ | ☐ | ☐ | ☐ |
| 1. If I were to temporarily stop working due to illness, I would feel guilty about increasing the workload for my colleagues. | ☐ | ☐ | ☐ | ☐ | ☐ |
| 1. Having the role of doctor could become a barrier if I should ever have to adopt the role of patient (in case of illness; such as treating myself, taking a few days sick leave, seeing a doctor, etc.). | ☐ | ☐ | ☐ | ☐ | ☐ |
| 1. It is not comfortable for a doctor to have to be the patient and take on the role of the sick person. | ☐ | ☐ | ☐ | ☐ | ☐ |
| **Physician Stigma towards Burnout** | | | | | |
| 1. Most doctors would consider the experience of occupational stress or burnout as a sign that the doctor is not "right" for the profession. | ☐ | ☐ | ☐ | ☐ | ☐ |
| 1. Most doctors would consider a doctor who is experiencing occupational stress or burnout as too sensitive or weak. | ☐ | ☐ | ☐ | ☐ | ☐ |
| 1. I would try to distance myself from a doctor who is experiencing occupational stress or burnout. | ☐ | ☐ | ☐ | ☐ | ☐ |
| 1. I would have reservations about working with a doctor who is experiencing difficulties with occupational stress or burnout. | ☐ | ☐ | ☐ | ☐ | ☐ |

**Legend Table K**: To obtain a score for each dimension separately, we averaged the items in each sub-dimension. To obtain an overall score for Harmful Dimensions of the Medical Culture (HDMC), we averaged all 21 items, where higher score indicates stronger internalization of the norms. This measure was developed in French language and has not yet been validated in an English-speaking sample. Items were back-translated from French to English.

**Additional references related to Table A.**

1. Henderson M, Brooks SK, del Busso L, Chalder T, Harvey SB, Hotopf M, et al. Shame! Self-stigmatisation as an obstacle to sick doctors returning to work: a qualitative study. BMJ Open. 2012;2: e001776. doi:10.1136/bmjopen-2012-001776

2. Mckevitt C, Morgan M. Illness doesn’t belong to us. J R Soc Med. 1997;90: 491–495. doi:10.1177/014107689709000907

3. Shanafelt TD. Physician Well-being 2.0: Where Are We and Where Are We Going? Mayo Clinic Proceedings. 2021;96: 2682–2693. doi:10.1016/j.mayocp.2021.06.005

4. Shanafelt TD, Schein E, Minor LB, Trockel M, Schein P, Kirch D. Healing the Professional Culture of Medicine. Mayo Clinic Proceedings. 2019;94: 1556–1566. doi:10.1016/j.mayocp.2019.03.026

5. Wainwright E, Fox F, Breffni T, Taylor G, O’Connor M. Coming back from the edge: a qualitative study of a professional support unit for junior doctors. BMC Med Educ. 2017;17: 142. doi:10.1186/s12909-017-0978-0

6. Clough BA, Ireland MJ, March S. Development of the SOSS-D: a scale to measure stigma of occupational stress and burnout in medical doctors. Journal of Mental Health. 2019;28: 26–33. doi:10.1080/09638237.2017.1370642

7. Brooks SK, Gerada C, Chalder T. Review of literature on the mental health of doctors: Are specialist services needed? Journal of Mental Health. 2011;20: 146–156. doi:10.3109/09638237.2010.541300

8. Brower KJ. Professional Stigma of Mental Health Issues: Physicians Are Both the Cause and Solution. Academic Medicine. 2021;96: 635–640. doi:10.1097/ACM.0000000000003998

9. Dyrbye LN, Leep Hunderfund AN, Winters RC, Moeschler SM, Vaa Stelling BE, Dozois EJ, et al. The Relationship Between Burnout and Help-Seeking Behaviors, Concerns, and Attitudes of Residents. Acad Med. 2021;96: 701–708. doi:10.1097/ACM.0000000000003790

10. Gerada C. Doctors, suicide and mental illness. BJPsych Bull. 2018;42: 165–168. doi:10.1192/bjb.2018.11

11. Kay M, Mitchell G, Clavarino A, Doust J. Doctors as patients: a systematic review of doctors’ health access and the barriers they experience. Br J Gen Pract. 2008;58: 501–508. doi:10.3399/bjgp08X319486

12. Shanafelt TD, West CP, Sinsky C, Trockel M, Tutty M, Wang H, et al. Changes in Burnout and Satisfaction With Work-Life Integration in Physicians and the General US Working Population Between 2011 and 2020. Mayo Clinic Proceedings. 2022;97: 491–506. doi:10.1016/j.mayocp.2021.11.021

13. Fältholm Y. “Patients, not doctors, get sick”: A study of fifteen Swedish physicians on long-term sick leave. International Journal of Qualitative Studies on Health and Well-being. 2007;2: 19–32. doi:10.1080/17482620601088277

14. Klitzman R. When doctors become patients. Oxford ; New York: Oxford University Press; 2008.

15. Thompson WT, Cupples ME, Sibbett CH, Skan DI, Bradley T. Challenge of culture, conscience, and contract to general practitioners’ care of their own health: qualitative study. BMJ. 2001;323: 728–731. doi:10.1136/bmj.323.7315.728

16. Daloz L. Epuisement professionnel et blessures de l’idéal. Réflexions cliniques sur la désillusion des soignants etla formation initiale. Pédagogie Médicale. 2007;8: 82–90. doi:10.1051/pmed:2007003

17. Clement S, Schauman O, Graham T, Maggioni F, Evans-Lacko S, Bezborodovs N, et al. What is the impact of mental health-related stigma on help-seeking? A systematic review of quantitative and qualitative studies. Psychol Med. 2015;45: 11–27. doi:10.1017/S0033291714000129

18. Onyura B, Bohnen J, Wasylenki D, Jarvis A, Giblon B, Hyland R, et al. Reimagining the Self at Late-Career Transitions: How Identity Threat Influences Academic Physicians’ Retirement Considerations. Academic Medicine. 2015;90: 794–801. doi:10.1097/ACM.0000000000000718

19. Crawshaw R. A Physician’s Oath for Self-Insight. Ann Intern Med. 1979;91: 648. doi:10.7326/0003-4819-91-4-648

20. Grassi L, McFarland D, Riba MB, editors. Depression, Burnout and Suicide in Physicians: Insights from Oncology and Other Medical Professions. Cham: Springer International Publishing; 2022. doi:10.1007/978-3-030-84785-2

21. Parsa-Parsi RW. The Revised Declaration of Geneva: A Modern-Day Physician’s Pledge. JAMA. 2017;318: 1971. doi:10.1001/jama.2017.16230

22. Adams EFM, Lee AJ, Pritchard CW, White RJE. What Stops Us From Healing the Healers: a Survey of Help-Seeking Behaviour, Stigmatisation and Depression Within the Medical Profession. Int J Soc Psychiatry. 2010;56: 359–370. doi:10.1177/0020764008099123

23. Brewin CB, Firth-Cozens J. Dependency and self-criticism as predictors of depression in young doctors. Journal of Occupational Health Psychology. 1997;2: 242–246. doi:10.1037/1076-8998.2.3.242

24. Enns MW, Cox BJ, Sareen J, Freeman P. Adaptive and maladaptive perfectionism in medical students: a longitudinal investigation. Med Educ. 2001;35: 1034–1042. doi:10.1046/j.1365-2923.2001.01044.x

25. Farrar C. Exploration of Physician Attitudes about Self-Care and Personal Mental Health: Possible Barriers to Physician Well Being. Dissertation, National Louis University. 2020.

26. Fox F, Harris M, Taylor G, Rodham K, Sutton J, Robinson B, et al. What happens when doctors are patients? Qualitative study of GPs. Br J Gen Pract. 2009;59: 811–818. doi:10.3399/bjgp09X472872

27. Walsh G, Hayes B, Freeney Y, McArdle S. Doctor, how can we help you? Qualitative interview study to identify key interventions to target burnout in hospital doctors. BMJ Open. 2019;9: e030209. doi:10.1136/bmjopen-2019-030209
