# Supplementary material for: Harmful dimensions of medical culture in relation to physician burnout: A cross-sectional study
Source: PLOS Ment Health. 2025 Apr 17;2(4):e0000301. doi: 10.1371/journal.pmen.0000301 (PMC12798346; doi:10.1371/journal.pmen.0000301)
Supplement: S1 Fig — Fig A. CFA model 1. First-order model of eight distinct but correlated factors as extracted from the EFA, representing the concepts of Work Priority Strain; Existential Significance of Being a Physician; Physician’s Moral Obligation to Patients and Colleagues; Colleagues’ Stigma towards PB; Personal Stigma towards PB; Physician’s Discomfort with Patient Role; Physician’s Central Identity Role; and the Sacrificial Nature of Medical Practice and their indicators of 24 items. In this figure, the top left column represents the error (e) terms in the confirmatory factor analysis. The second column (rectangles) represents the items, the third column (arrows) represents the item loadings from the items to the first order latent factors, the fourth column (ovals) represents the first order latent factors found in the EFA, and the last column (arrows) represents the inter-factor correlations. Fig B. CFA model 2. First-order model of seven distinct but correlated factors extracted from the EFA, representing the concepts of Work Priority Strain; Physician’s Moral Obligation to Patients and Colleagues; Colleagues’ Stigma towards PB; Personal Stigma towards PB; Physician’s Discomfort with Patient Role; Physician’s Central Identity Role; and the Sacrificial Nature of Medical Practice and their indicators of 21 items. The top left column represents the error (e) terms in the confirmatory factor analysis. The second column (rectangles) starting from the left represents the items. The third column (arrows) represents the item loadings from the items to the first order latent factors. The fourth column (ovals) represents the first order latent factors found in the EFA. The last column (arrows) represents the inter-factor correlations. Fig C. CFA model 3. The second-order one-factor model in which the latent factors of Model 2 are supposed to measure harmful dimensions of the medical culture, loading onto a general factor for Harmful Dimensions of the Medical Culture (HDMC). The top l [file pmen.0000301.s002.docx]

**
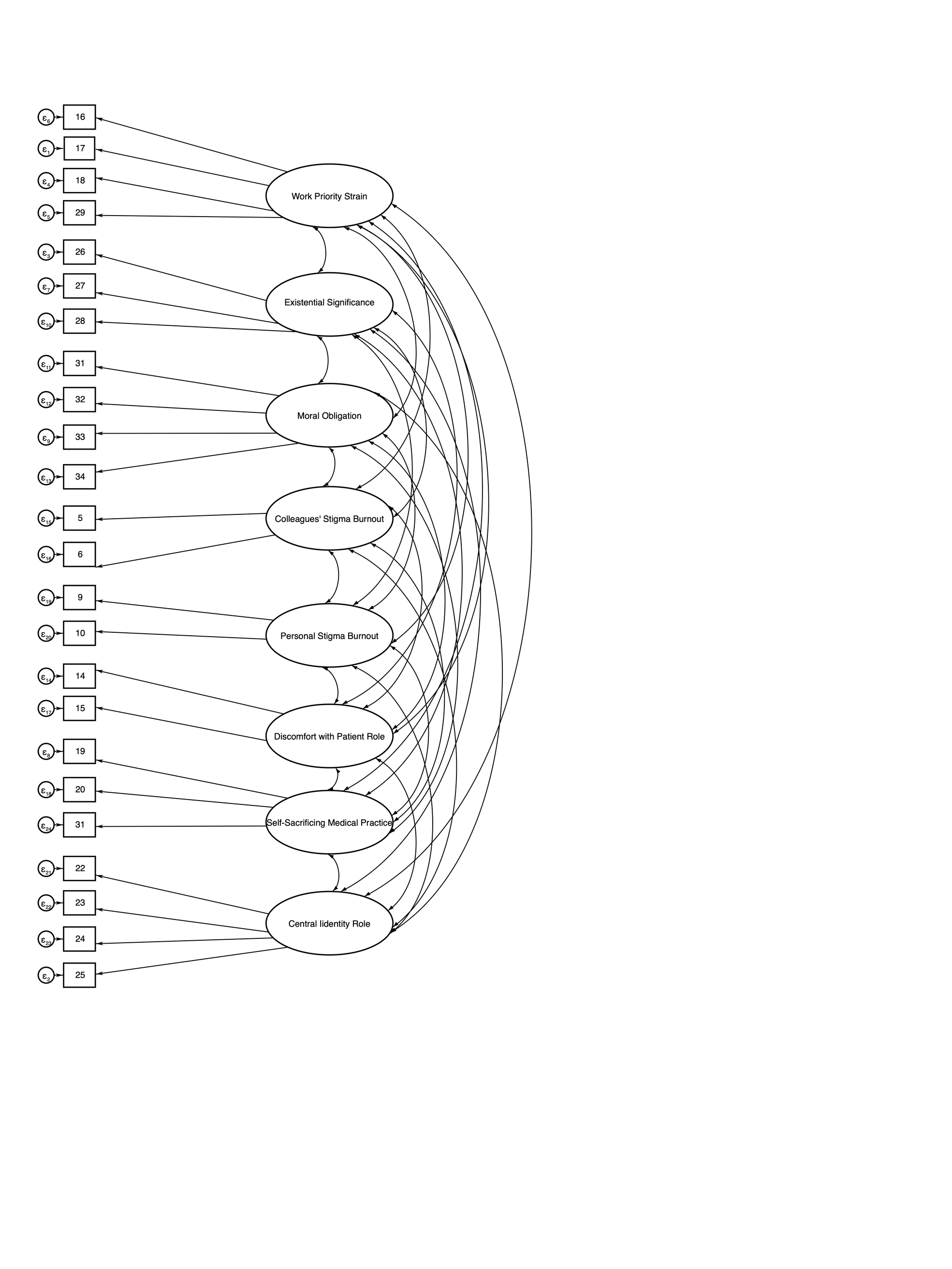
Fig A. CFA model 1.**

**Legend Fig A**: First-order model of *eight* distinct but correlated factors as extracted from the EFA, representing the concepts of Work Priority Strain; Existential Significance of Being a Physician; Physician’s Moral Obligation to Patients and Colleagues; Colleagues’ Stigma towards PB; Personal Stigma towards PB; Physician’s Discomfort with Patient Role; Physician’s Central Identity Role; and the Sacrificial Nature of Medical Practice and their indicators of 24 items. In this figure, the top left column represents the error (e) terms in the confirmatory factor analysis. The second column (rectangles) represents the items, the third column (arrows) represents the item loadings from the items to the first order latent factors, the fourth column (ovals) represents the first order latent factors found in the EFA, and the last column (arrows) represents the inter-factor correlations.

**
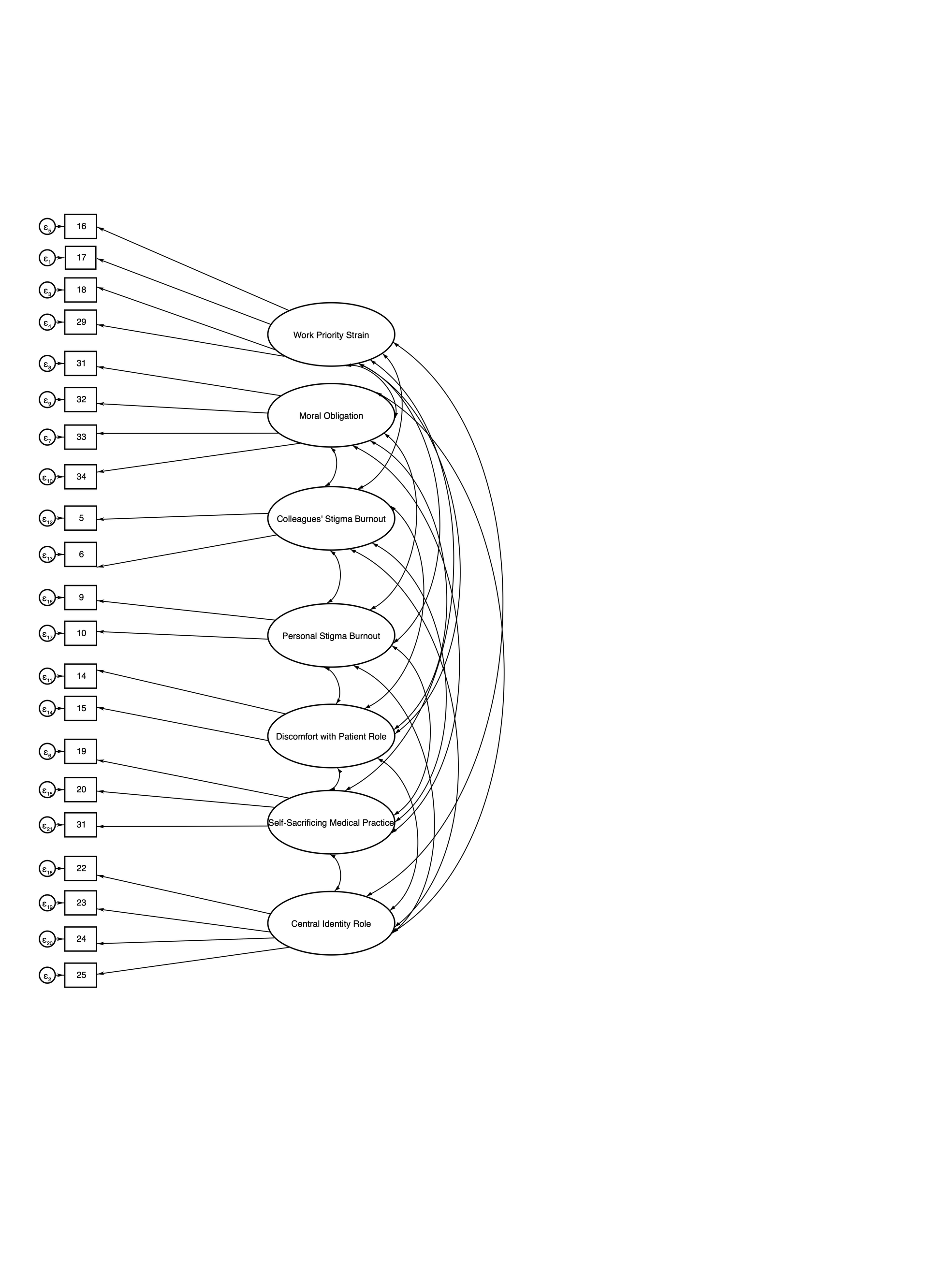
 Fig B. CFA model 2.**

**Legend Fig B**: First-order model of *seven* distinct but correlated factors extracted from the EFA, representing the concepts of Work Priority Strain; Physician’s Moral Obligation to Patients and Colleagues; Colleagues’ Stigma towards PB; Personal Stigma towards PB; Physician’s Discomfort with Patient Role; Physician’s Central Identity Role; and the Sacrificial Nature of Medical Practice and their indicators of 21 items. The top left column represents the error (e) terms in the confirmatory factor analysis. The second column (rectangles) starting from the left represents the items. The third column (arrows) represents the item loadings from the items to the first order latent factors. The fourth column (ovals) represents the first order latent factors found in the EFA. The last column (arrows) represents the inter-factor correlations.


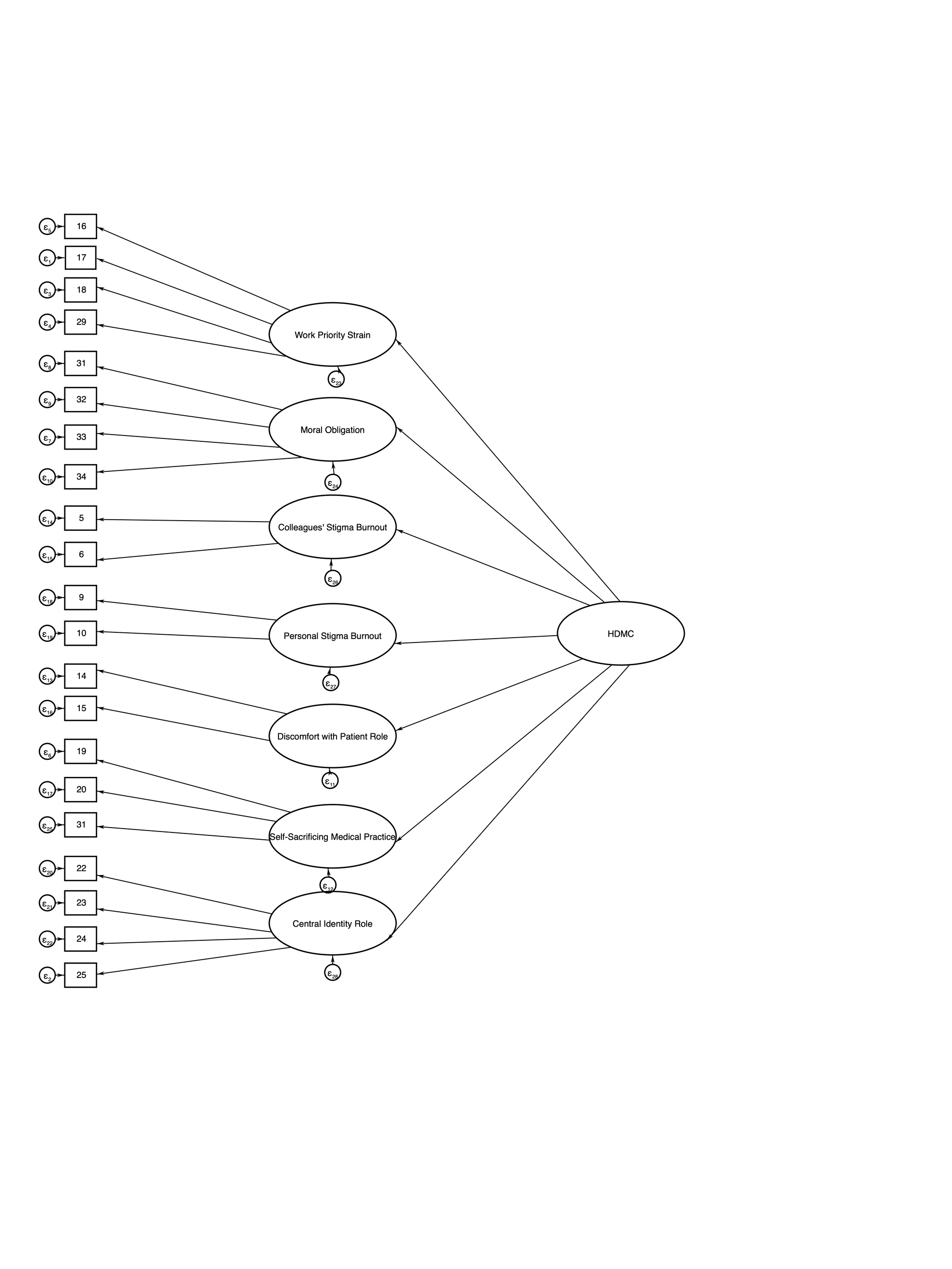
 **Fig C. CFA model 3.**

**Legend Fig C:** The second-order one-factor model in which the latent factors of Model 2 are supposed to measure harmful dimensions of the medical culture, loading onto a general factor for Harmful Dimensions of the Medical Culture (HDMC). The top left column represents the error (e) terms the confirmatory factor analysis. The second column (rectangles) starting from the left represents the items. The third column (arrows) represents the item correlations with the latent constructs for each item, the fourth column (ovals) represents the first-order latent factors found in the EFA, the fifth column (arrows) represents the loadings from the first order latent factors to the second-order latent HDMC factor, and the final column (ovals) represents the second order latent factor HDMC.

**
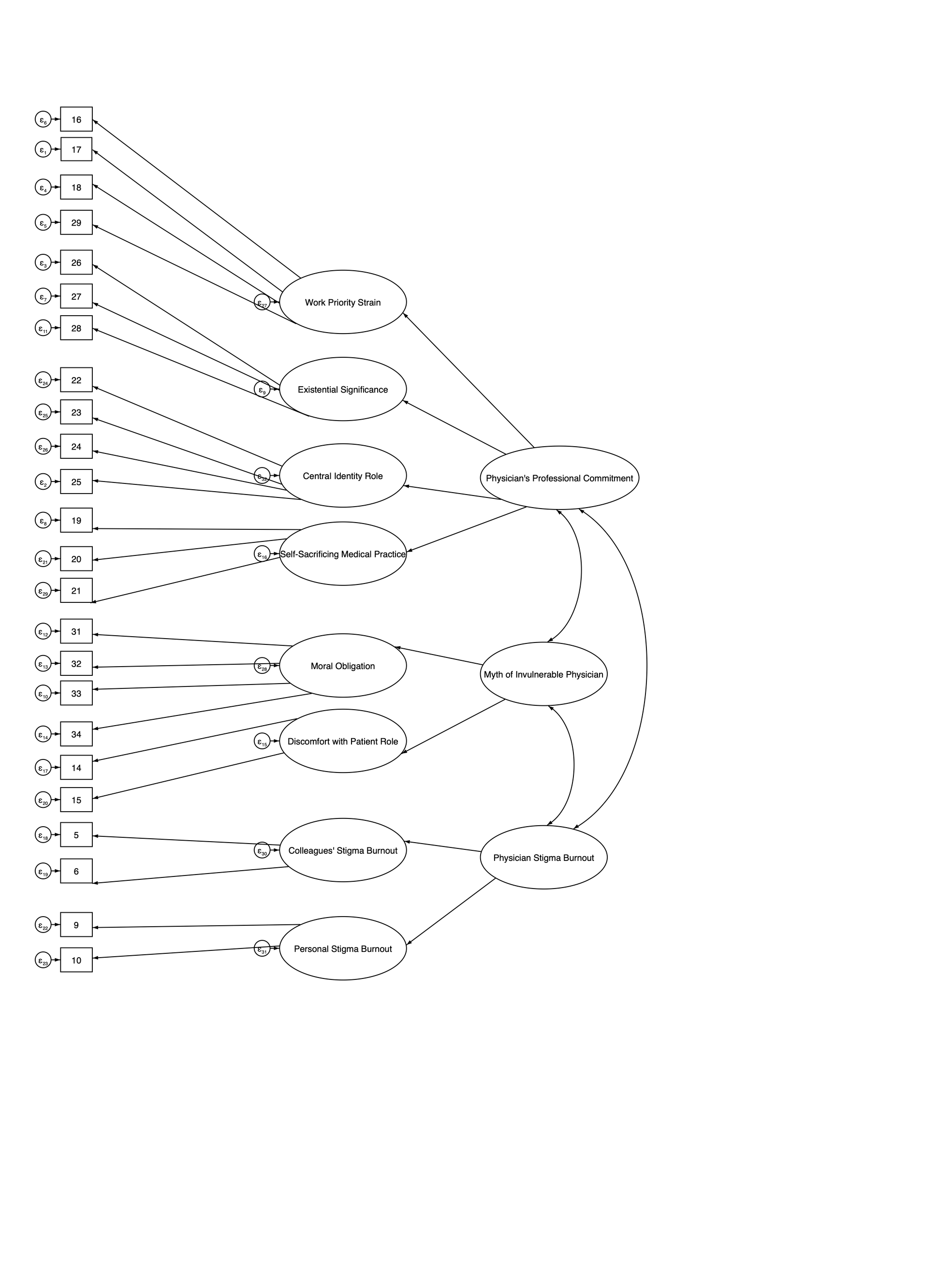
 Fig D. CFA model 4.**

**Legend Fig D**: Second-order hierarchical model where the *eight* initial factors of model 1 load onto three second-order dimensions (Physician’s Professional Commitment; The Myth of the Invulnerable Physician; and Physician Stigma towards Burnout – *with* the latent factor of Existential Significance of Being a Physician). The top left column represents the error (e) terms in the confirmatory factor analysis. The second column (rectangles) starting from the left represents the 24 items. The third column (arrows) represents the item correlations with the latent constructs for each item, the fourth column (ovals) represents the first-order latent factors found in the EFA, the fifth column (arrows) represents the loadings from the first-order latent factors to the second-order latent dimensions, and the final column (ovals) represents the second-order latent factors with inter-factor correlations (arrows).

**
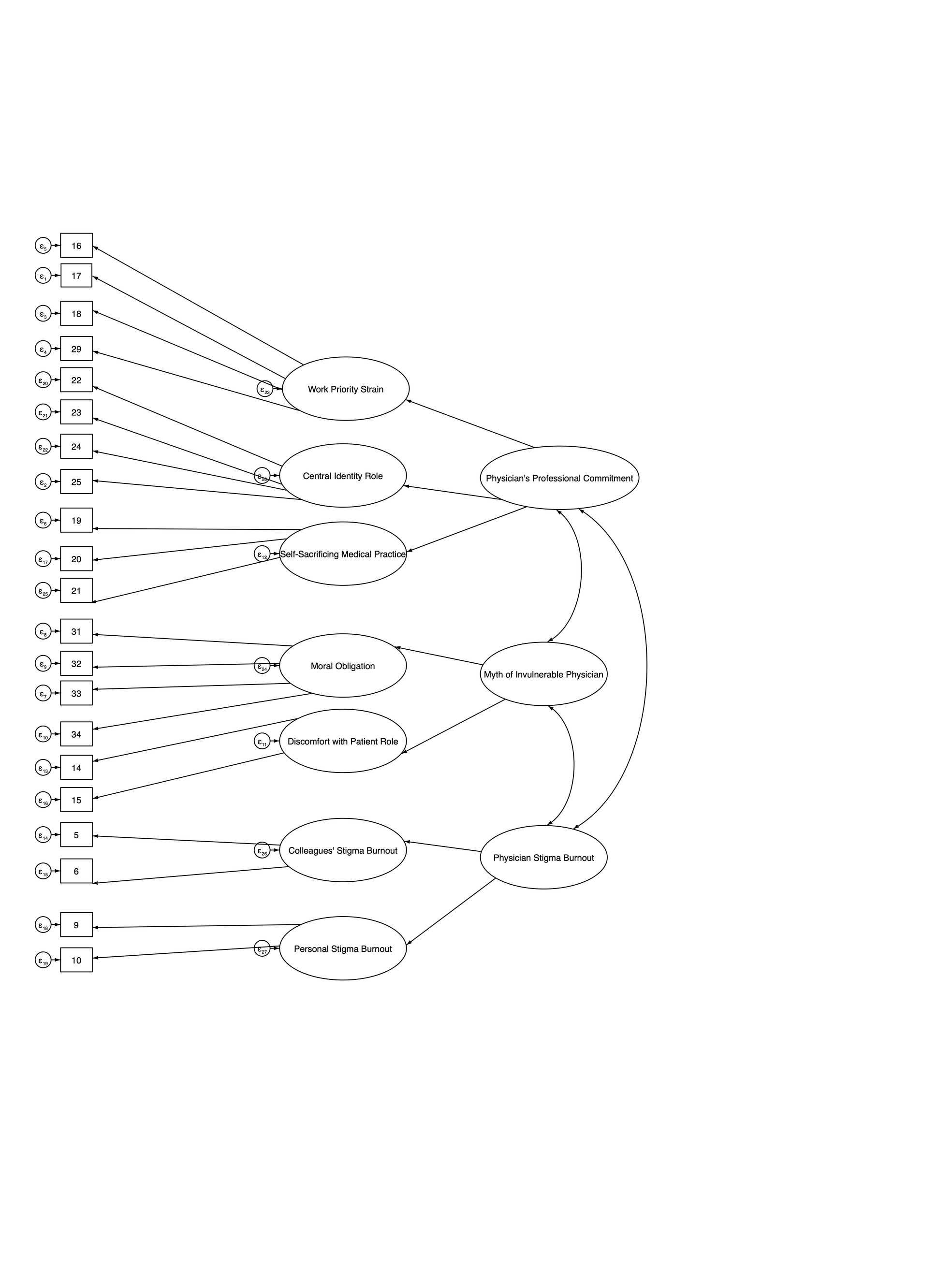
 Fig E. CFA model 5.**

**Legend Fig E:** Second-order hierarchical model where *seven* factors of model 1 load onto three second-order dimensions (Physician’s Professional Commitment; The Myth of the Invulnerable Physician; and Physician Stigma towards Burnout – *without* the latent factor of Existential Significance of Being a Physician). The top left column represents the error (e) terms in the confirmatory factor analysis. The second column (rectangles) starting from the left represents the 21 items. The third column (arrows) represents the item correlations with the latent constructs for each item, the fourth column (ovals) represents the first-order latent factors found in the EFA, the fifth column (arrows) represents the loadings from the first-order latent factors to the second-order latent dimensions, and the final column represents the second-order latent dimensions (ovals) with inter-factor correlations (arrows).
