# Supplementary material for: Harmful dimensions of medical culture in relation to physician burnout: A cross-sectional study
Source: PLOS Ment Health. 2025 Apr 17;2(4):e0000301. doi: 10.1371/journal.pmen.0000301 (PMC12798346; doi:10.1371/journal.pmen.0000301)
Supplement: S1 Text — Methods. (DOCX) [file pmen.0000301.s003.docx]

**S1 Text (Methods).**

**Sample size calculation.**

The sample size for this study was determined based on the need to conduct Exploratory Factor Analyses (EFA), correlational analyses, and linear multiple regressions, following guidelines for each statistical procedure. For the EFA, which involved a total of 35 items and 3 expected factors, the sample size required for good factor recovery was estimated at 175 participants, based on the guidelines of Child [1] and Watkins [2]. Since multiple analyses were to be conducted, we planned to recruit up to 50 additional participants beyond this minimum estimate to account for potential exclusions and dropout. For the correlational analyses, G*Power was used to determine the sample size required to achieve a power of 0.90 for detecting a medium effect size (r = 0.2). Based on this calculation, the minimum sample size required was 211 participants. The sample size for the linear multiple regressions could not be precisely predicted before the EFA results, as the number of factors representing the professional norms of the medical culture was not yet known. However, based on our expectations of having 3 independent factors (representing cultural dimensions) plus the control variable (social desirability), the sample size for linear regression with 4 independent variables was estimated at 124 participants, using G*Power with an expected small to medium effect size (F² = 0.13).Given the combination of these analyses, the minimum required sample size for the study was estimated to be 225 participants, and we aimed to recruit up to 550 participants to account for possible exclusions.

**Missing values and drop-out analyses.**

For ethical reasons and as some questions in the survey might be sensitive for participants, participants were not required to answer to all questions, leading the door open to missing values. A total of 1292 physicians connected to the online survey, of which 1238 provided consent and 1002 provided complete data for the cultural items. Visual inspection of the missing values pattern indicated that most missing values were drop out cases throughout the survey. As, for the factorial analyses on cultural items, we were interested in participants who had no missing values on the items reflecting cultural dimensions (N = 1002), we tested whether differences existed between those participants and participants who had dropped out on the cultural items (N = 236), using t-tests and chi^2^ tests. They were found to be similar on all variables of interest, except for the status of the physician (being an attending physician or being a resident). Indeed, there was a significant association between the status of the physician participant and whether or not he/she would drop out on the cultural items χ^2^(4) = 5.80, *p* = 0.02. The odds of participants dropping out was 1.53 times higher if the participant was a resident physician than if the participant was an attending physician. However, the associated effect size was small (φ = .070, *p*. = 0.02). We also tested whether differences existed between individuals who provided complete data on all items of interest for the present analyses (i.e. 35 items of culture and 12 items measuring burnout) (N = 973) and individuals who provided complete data on the cultural items solely (N = 1002) on demographic and work-related variables (age, gender, seniority, number of hours worked per week, status of the physician, specialty) as well as on burnout using t-tests and chi^2^ tests. They were found to be strictly similar regarding those variables. Statistical analyses were therefore conducted on the sample of 1002 to maximize the statistical power of the factorial analyses. Characteristics of non-responders cannot be given or compared to characteristics of the responders as the sample used in the present study is a convenience sample.

**Exploratory factor analysis (EFA).**

By means of cross-validation, the total sample was randomly split in two samples of 501 participants for either EFA or CFA. The comparability of the two sub-samples was assessed. Then, exploratory factor analysis was first conducted using principal axis factoring (PAF) and direct oblimin rotation on the 35 items (N = 501) using SPSS. EFA permits to explore the number of meaningful underlying factors and to retain a pool of items from the initial total number of items. The Kaiser-Meyer-Olkin (KMO) measure of sampling adequacy and Bartlett’s test of sphericity assessed suitability of our data. Item loadings of ≥ 0.40 and communalities > 0.20 were required, cross-loading items were defined as items loading ≥ 0.30 on two or more factors. Those items were considered unstable and were therefore removed. The proportion of total variance explained (TVE) by the factors needed to be ≥ 50%. To determine the number of factors to retain, we relied on various methods: Kaiser’s criterion, the scree plot and the parallel analysis [2–5]. The KMO measure of sampling adequacy of the first EFA conducted on the initial 35-item pool was high (0.85) and Bartlett’s test of sphericity was significant, ­χ^2^(595) = 6723.81, p<0.001, suggesting the item pool was suitable for EFA. According to Kaiser’s criterion, ten factors were extractable (51.1% of TVE). However, the scree plot suggested a seven-factor structure (46.37% of TVE) and the parallel analysis (considered as the most reliable method for extraction) suggested an eight-factor structure (48.24% of TVE). Several analyses were run until a stable solution was found, removing items one by one until the solution satisfied all requirements mentioned above. ; Items 3 and 13 exhibited communalities < 0.20 and were removed. The PAF analysis was rerun among 33 items and the number of factors to extract was set to 8, following the results of the parallel analysis (note that various analyses with 7, 8 and 9 factors to extract were tested. Solutions with 8 factors to extract were the most satisfactory as solutions with 7 or 9 factors showed signs of either under- or overextraction). Items 1, 2, 4, 7 and 8 presented no loading ≥ 0.40 and were therefore removed. An eight-factor PAF analysis was rerun on the remaining 28 items and items 11 and 12 were then removed as they presented no loading ≥ 0.40. Lastly, items 29 and 35 were also removed for the same reasons. The remaining 24 items were again submitted to PAF with 8 factors to extract. The KMO measure of sampling adequacy of this EFA was high (0.84) and Bartlett’s test of sphericity was significant, ­χ^2^(276) = 5069.83, p<0.000. The solution accounted for 59% of item variance and *a* *simple structure was obtained* (with *no* cross-loading items). Communalities for all items were satisfactory (ranging between 0.45 and 0.82). Intercorrelations between the factors were low to medium, confirming the interest of using a Direct Oblimin rotation. Sensitivity analyses were conducted, as we compared if the solution obtained was the same without and with deleting outliers found in our sample. The structure was unchanged. This acted as a supplementary criterion for the stability of the model.

**Confirmatory factor analysis (CFA).**

A confirmatory factor analysis with maximum likelihood estimation method was then performed in the second subsample to cross-validate the structure emerging from the EFA (using STATA). CFA permits to cross-validate the factor structure that emerged from EFA, but also to compare this structure to other models based on theoretical and/or statistical grounds. A total of 5 models (outlined in S1 through S5 figures and described in S5 table) were compared using different goodness-of-fit indices to determine the statistical acceptability of the models and to retain the best-fitting model: the chi-square indices (χ2) [6], the root mean square error of approximation (RMSEA), the standardized root mean square residual (SRMR), the comparative fit index (CFI), and the Tucker-Lewis index (TLI) [7]. Acceptable fit criteria included RMSEA and SRMR ≤ 0.08, CFI and TLI > 0.90 [6,7].

**Internal consistency of the scales.**

Final scales representing the dimensions were created on SPSS, based on the mean scores of the items of each dimension. Alphas of minimum 0.60 were deemed as acceptable regarding internal consistency for scales with 10 items or less. For scales/subscales with two items, interitem correlations were computed and values of minimum 0.50 were desirable [2].

**Multivariable regression analyses.**

Multivariable regression analyses to predict burnout based on the HDMC were conducted on SPSS (linear regression – two blocks – enter method), adjusted after controlling for well-known covariates of burnout. Cases with missing values were excludes listwise.

Among our variables of interest, the following personal and professional characteristics were significantly associated to burnout in the sample: age (*ρ* = -0.10, p= 0.002), years in practice (*ρ* = -0.12, p< 0.001), the status of the physician (*U* = 76196.00, *p* = <0.001; residents had higher median scores of burnout, Mdn = 2.33, IQR = 0.75), the parental status (*U* = 105642.00, *p* = 0.002; physicians who had no children had higher median scores of burnout, Mdn = 2.33, IQR = 0.67). Regarding gender, a Kruskal-Wallis test showed that there was a statistically significant difference in burnout scores between the three categories of gender χ^2^(2) = 23.76, p <0.001. Post-hoc pairwise comparisons using the Bonferroni correction revealed a significant difference in burnout scores between male and female with female reporting higher burnout scores adjusted (adjusted p = 0.000), a marginally significant difference in burnout scores between males and the “other” category (p = 0.051) and no significant difference in burnout scores between females and the “other” category (p = 0.15). Note that the category “other” had a very small sample size (N = 2). The variables relationship status (being single vs being in a relationship), hours working estimated per week and specialty (being a general practitioner vs being a specialist) were not significantly associated to burnout in our sample.

To avoid multicollinearity between the variables age and years in practice, we selected years in practice and excluded age from the regression analysis. All other covariates were included as controls. Two physicians identified as belonging to the "other" gender category had significantly higher burnout scores. Due to the small number of physicians in this gender category, we decided to exclude these two cases from the regression analysis to ensure the stability and reliability of our statistical estimates. In the second step, the three second-order HDMC dimensions and the factor Existential Significance were added to the regression model using the enter method.

Following Field's guidelines regarding casewise diagnostics in regressions [4], 38 cases with standardized residuals less than -2 or greater than 2 were identified as outliers. However, the results did not significantly change when these outliers were excluded, so we decided to retain them in the analysis. Other assumptions were verified. S9 table presents the results with controls for social desirability, along with a brief interpretation of these findings. The interplay between the characteristics of the physicians, their burnout scores, and the internalization of the dimensions of medical culture will be further discussed in a forthcoming paper.

**Additional references with S1 Methods.**

1. Child D. The essentials of factor analysis. 3rd ed. London ; New York: Continuum; 2006.

2. Watkins MW. A Step-by-Step Guide to Exploratory Factor Analysis with SPSS. 1st ed. New York, NY : Routledge, 2021.: Routledge; 2021. doi:10.4324/9781003149347

3. Tabachnick BG, Fidell LS. Using multivariate statistics. Pearson new international edition, sixth edition. Harlow: Pearson; 2014.

4. Field A. Discovering statistics using SPSS: and sex, drugs and rock “n” roll. 3rd ed. Los Angeles: SAGE Publications; 2009.

5. Hayton JC, Allen DG, Scarpello V. Factor Retention Decisions in Exploratory Factor Analysis: a Tutorial on Parallel Analysis. Organizational Research Methods. 2004;7: 191–205. doi:10.1177/1094428104263675

6. Byrne BM. Structural equation modeling with LISREL, PRELIS, and SIMPLIS: basic concepts, applications, and programming. Online-ausg. Mahwah, N.J: L. Erlbaum Associates; 1998.

7. Hu L-T, Bentler PM. Evaluating model fit. Structural equation modeling: Concepts, issues, and applications. London, UK: Sage Publications, Inc.; 1995. pp. 76–99.
