# Supplementary material for: Harmful dimensions of medical culture in relation to physician burnout: A cross-sectional study
Source: PLOS Ment Health. 2025 Apr 17;2(4):e0000301. doi: 10.1371/journal.pmen.0000301 (PMC12798346; doi:10.1371/journal.pmen.0000301)
Supplement: Attachment — Submitted filename: Response to Reviewers.docx [file pmen.0000301.s005.docx]

We sincerely thank the reviewers for their constructive feedback, which has significantly contributed to enhancing the quality and clarity of our manuscript. We especially appreciate the reviewers' recognition of the relevance of our study and the innovative aspects of our approach. Based on their comments, we have made substantial improvements to the manuscript, including clarifying the study's primary objectives, incorporating additional methodological details, and revising the tables for greater clarity. Please find bellow a point-by-point reply to the reviewers’ comments (in blue) as well as references to the corresponding changes that we made in the manuscript (in orange).

Reviewer #1

This study addresses the contribution of medical culture to physician burnout. Few data exist on this specific question, which is a relevant aspect in this field.

Title and Abstract: The study’s design is stated in the title, and the abstract provides a balanced summary.

Introduction: Previous literature was cited and treated fairly. The scientific background reports on recent studies. The specific objectives are stated.

Methods: Ethics and instruments are reported.

The study was carried out in a French-speaking country. Which validated translations of the instruments were used?

Reply: We thank the reviewer for this comment, and have specified the version of the Burnout Assessment Tool (BAT-12) used in the study. Specifically, we used the French version of the BAT-12, as proposed by the original authors of the scale (Schaufeli et al., 2019). This version, titled the “*version travail du BAT*,” is validated and widely used in French-speaking contexts. We have updated the manuscript to explicitly mention this information, including the appropriate citation:

Changes in the manuscript: see p. 5: “We used the French version of the BAT-12; as proposed by the original authors of the scale [49].”

Added reference: see p. 22: 49. Schaufeli WB, De Witte H, Desart S, Securex. Handleiding Burnout Assessment Tool (BAT). KU Leuven, Belgium; 2019. Available: https://burnoutassessmenttool.be/wp-content/uploads/2020/08/BAT-Françias-France.pdf

Population: The eligibility criteria and sources of participant selection are given. Convenience sampling is stated.

Period of recruitment is not given.

Reply: Thank you for highlighting this point. The recruitment period for the study was from September 5 to November 5, 2023, which corresponds to the data collection phase. This information was included in the manuscript, but we realize it may not have been sufficiently emphasized. To ensure clarity, we have revised the relevant section to explicitly state the recruitment period alongside the description of data collection:

Changes in the manuscript: see pp. 4-5: “Recruitment of participants occurred iteratively during the data collection phase, which lasted from September 5 to November 5, 2023.”

Statistical analyses are clearly described.

Outcomes, potential confounders, and effect modifiers are not clearly defined, nor are potential sources of bias.

Reply: We thank the reviewer for raising this important point, which allowed us to clarify and improve the manuscript. Below, we provide additional details regarding the study design and analyses. Along with each of these clarifications, we indicate in orange how they have been incorporated into the manuscript to address the reviewer’s concerns and enhance our presentation of the study design and statistical analyses.

In our study, the **outcome** was physician burnout, which served as the dependent variable in our analyses. The medical culture, which we investigated using a psychometric instrument, was considered the independent variable or predictor.

Changes in the manuscript: see p. 6: “Physician burnout was defined as the outcome (dependent variable). The independent variable, or predictor, was the structure of medical culture.”

Personal and professional characteristics known to influence burnout were considered **potential confounders** and were included in the first step of the hierarchical regression model. These included gender, relationship status, parental status, physician status (resident vs attending), specialty (generalist vs specialist), years in practice, and weekly working hours. We have endeavored to distinguish more clearly these concepts in the revised manuscript, especially in the Methods section, where we explicitly define the outcomes, predictors, and potential confounders.

Changes in the manuscript: see p. 7: “The hierarchical regression analysis included the following potential confounders in the first step of the model: gender, relationship status, parental status, physician status (resident vs attending), specialty (generalist vs specialist), years in practice, and weekly working hours, as these personal and professional characteristics are known to be associated with PB [31,35,37].”

While **effect modifiers** were not directly investigated in this study, the association between burnout and medical culture dimensions was assessed after adjusting for potential confounders, ensuring robustness of the observed relationships. We discussed in the limitations and future directions section of the manuscript how future studies should explore how the various personal and socioprofessional characteristics of the physicians could have influenced the endorsement of the HDMC, and how such characteristics moderate the association between the HDMC and PB.

Finally, **potential sources of bias** of this study are addressed in the discussion section of the manuscript. As the study used a convenience sample, we were unable to estimate the net response rate for participation, which may raise caveats about the generalizability of our findings. For ethical reasons, and as some questions in the survey might be sensitive for participants, we did not require the participants to answer to all questions, thus leaving the door open to missing values. The missing values primarily resulted from participant dropout during the survey. Analyses comparing participants with complete data (N = 1002) to those with missing data (N = 236) showed only minor differences: residents were slightly more likely to drop out (χ2(4) = 5.80, p = 0.02, φ = 0.070), with a small effect size. We also tested whether there were differences between the individuals who provided complete data on all items of interest for the present analyses (i.e. 35 items of culture and 12 items measuring burnout) (N = 973) and those who provided complete data solely on the cultural items (N = 1002) on demographic and work-related variables (age, gender, seniority, number of hours worked per week, status of the physician, specialty), as well as on burnout using t-tests and χ2 tests. Results were essentially identical regarding those variables. These sources of bias are now detailed in the revised manuscript:

Changes in the manuscript: see p. 6: “Missing values were primarily due to participant dropout during the survey. Participants with complete data on the cultural items (N = 1002) were compared to those with missing data (N = 236), revealing only small differences, notably a higher likelihood of survey dropout among residents (χ2(4) = 5.80, p = 0.02, φ = 0.070). Potential differences were also examined between individuals who provided complete data on all items of interest (35 cultural items and 12 burnout items, N = 973) and those who provided complete data only on the cultural items (N = 1002) on demographic and work-related variables (age, gender, seniority, number of hours worked per week, status of the physician, specialty) as well as on burnout, using t-tests and χ2 tests. No significant differences were found between these groups, indicating that they were comparable with respect to these variables. To maximize statistical power, analyses were conducted on the largest possible samples.”

We took steps to ensure the stability and robustness of the factor structure. Sensitivity analyses, including the removal of outliers and adjustments to the factor retention process (such as testing different criteria for factor loadings and examining solutions with varying numbers of factors to extract), confirmed the stability of the resulting factor structure. These steps were already detailed in the methods section of the supplementary material, but are now also summarized in the revised manuscript:

Changes in the manuscript: see p. 7: “We performed sensitivity analyses to test the stability of factor solutions (e.g., after removing outliers or changing factor retention criteria). These analyses supported the robustness of the identified factor structure.”

Tables are informative. I guess it’s only in the French-speaking part of Switzerland that participants were included (if so specify).

Reply: The inclusion criteria specified that participants had to be French-speaking physicians with a current clinical practice. While we initially planned only recruiting only physicians practicing in Belgium, the online survey (which was widely shared through social media and word of mouth) allowed for the inclusion of French-speaking physicians from other countries as well. We have added this information in the manuscript to ensure clarity:

Changes in the manuscript: see p. 5: “The survey was also disseminated on social media networks and via word of mouth. While the initial recruitment strategy was focused on physicians practicing in Belgium, this online dissemination allowed for the participation of French-speaking physicians from other countries as well. Participants were not excluded based on their country of residence, as long as they met the inclusion criteria.”

Discussion: Key results are summarized, and limitations are discussed.

I didn’t see the Funding information statement.

Reply: We thank the reviewer for raising this important point. The request for the funding information statement arose during the online submission process, and, as per the journal’s guidelines, it will be included in the typeset version of the manuscript in the event of acceptance for publication. For clarification, we made the following declaration in the online submission process:

“EB is a research fellow of the Fonds de la Recherche Scientifique – FNRS (Belgium), grant number 40010334. This funding agency did not exert any influence or censorship on the present work.”

The authors could consider using the STROBE checklist for cross-sectional studies and specify this in the manuscript.

Reply: We thank the reviewer for this suggestion. We have reviewed the STROBE checklist for cross-sectional studies, and have ensured that our manuscript aligns with its recommendations. We have added a statement in the manuscript to acknowledge this, and confirm that we followed the checklist when preparing our manuscript:

Changes in the manuscript: see p. 4: “This report complies with the Checklist for Reporting of Survey Studies [45] and follows the Strengthening the Reporting of Observational Studies in Epidemiology (STROBE) reporting guideline for cross-sectional studies.”

Added reference: see p. 22: 45. Von Elm E, Altman DG, Egger M, Pocock SJ, Gøtzsche PC, Vandenbroucke JP, et al. The Strengthening the Reporting of Observational Studies in Epidemiology (STROBE) Statement: Guidelines for Reporting Observational Studies. PLoS Med. 2007 Oct 16;4(10):e296).

The use of data from different countries (different cultures that may impact the local medical culture) could be discussed, as could the different types of hospitals and specialties. This could be discussed in the Discussion section.

Reply: We thank the reviewer for this thoughtful comment. We agree that the use of data from French-speaking physicians across different countries, as well as the potential influence of hospital type and specialty on medical culture and burnout, warrants discussion.

Regarding geographic variation, while our study primarily included Belgian physicians, our online survey methodology did not exclude French-speaking physicians from other countries. This choice acknowledges the possibility of differences in general culture, healthcare systems, and local medical cultures between countries, which might influence the results. As such, this element of design could be considered a limitation of the study. However, we rather view this as a potential strength, as it allows exploration beyond a single national context, in contrast to many studies conducted exclusively in single-country settings. This approach supports the hypothesis that, despite local variations, a shared medical culture transcending national boundaries could exist, shaped by societal expectations placed upon physicians, medical training, and professional practice, and that this culture contributes to burnout. Regarding hospital type and specialty, we acknowledge that specific workplace settings (e.g., private vs. public hospitals, academic vs. non-academic institutions) and specialties may introduce meaningful variation in medical culture and its association with burnout. For example, physicians in academic hospitals may experience additional pressures related to teaching and research, while the practical realities and stressors of certain specialties may also modulate the impact of medical culture on burnout. Future research could investigate these differences more thoroughly. To address the reviewer’s concern, we have added a discussion of these points to the manuscript, as suggested:

Changes in the manuscript: see p. 17: “While the present study primarily included Belgian physicians, the inclusion criteria allowed participation from French-speaking physicians practicing in other countries. This inclusion introduced the possibility of variations in general culture, healthcare systems, and local medical cultures between countries, which could have influenced our findings. While this diversity could be viewed as a limitation, as we were unable to account for potential differences linked to these contextual factors, in our assessment it represents a strength, as it permits exploration beyond a single national context. The consistent findings support the hypothesis that a shared medical culture exists across countries, characterized by professional norms and expectations instilled during medical training and professional practice, and that this culture significantly contributes to PB irrespective of national variations. […] However, our study did not explicitly investigate how medical culture and its association with burnout may vary according to various personal (e.g., personality, generation) and socio-professional (e.g., status, specialty, work setting) characteristics. For example, physicians in academic hospitals may face additional demands related to teaching and research, which might amplify certain aspects of medical culture, such as professional commitment [3,43]. Similarly, the realities of specific specialties may interact with cultural norms differently, thereby influencing their impact on burnout.”

Overall, the manuscript is well-organized and accessible to non-specialists.

Reviewer #2:

The article addresses harmful dimensions in medical practice and their association with burnout among physicians, bringing a current and relevant topic into focus through an extensive review of the literature on the collective and individual factors shaping attitudes toward the practice of medicine. The proposal to validate a psychometric instrument for assessing these dimensions is innovative and represents the study's strongest contribution.

However, there is a disconnect between the main objective of the article — the validation of the instrument — and the way it is presented. Although the study is fundamentally psychometric, it is described as an observational study, which may confuse readers and divert attention from its primary contribution. The cross-sectional data exploring the relationship between harmful dimensions and burnout are valuable for one stage of the instrument's validation, particularly for reliability analysis, but they are secondary to the study's main goal.

Reply: We thank the reviewer for this insightful comment. We agree that the study presents two complementary yet distinct aspects: (1) the psychometric exploration of the dimensions of medical culture through EFA and CFA, and (2) the investigation of the association between medical culture dimensions and burnout. While the primary goal of this study was to investigate the relationship between harmful dimensions of medical culture (HDMC) and physician burnout, the psychometric work emerged as a key methodological step to rigorously assess these dimensions. Although the psychometric work represents a significant contribution, this study was fundamentally intended to investigate the link between the harmful aspects of medical culture and physician burnout.

In response to this comment, we have clarified the study’s objectives and methodological scope throughout the manuscript. We have also explicitly addressed the psychometric contribution, while emphasizing its exploratory nature. These modifications aim to ensure that readers understand both the focus and the limitations of the psychometric work. We have updated the manuscript as follows to address these concerns.

In the introduction, we added the following paragraph:

Changes in the manuscript: see p. 4: “While this psychometric work represents an important methodological contribution of the study, our main goal was to examine the relationship between harmful aspects of medical culture and burnout.”

In the discussion, the following paragraph was added:

Changes in the manuscript: see pp. 18: “Future studies might also expand on the psychometric work initiated in this study. It would be particularly valuable to extend the examination of the criterion and predictive validity of the scale, notably by investigating its impact on variables related to physicians' health and well-being (e.g., depression, anxiety, reluctance to seek help, and self-medication), as well as team-level outcomes (e.g., quality of the work climate, interpersonal conflicts, workload distribution, and team collaboration) and patient-level factors (e.g., diminished empathy, medical errors, or, conversely, enhanced dedication to patient care and continuity of care). These prospects for future studies could provide a more comprehensive understanding of the impact of harmful dimensions of medical culture (HDMC), and illustrate how these dimensions may exert differential effects, including potentially positive outcomes in specific contexts.”

Specific Issues:

a) The introduction could be adjusted to better align with the journal's style. Beginning with a quote is not incorrect but may deviate from standard editorial practices. A more cohesive approach to introducing the problem is recommended.

Reply: We thank the reviewer for the suggestion to revise the introduction to better align with the journal’s style. While the opening quote was intended to illustrate the study’s thematic focus, we agree with the need to better align the manuscript’s introduction with standard editorial practices.

Changes in the manuscript: see p. 3: We have deleted the quote that was originally included in the beginning of the introduction, to provide a more direct and focused entry into the topic of physician burnout.

b) Following the STROBE guidelines, it is essential to present the context and participant scenario (number of potential participants by country). If a census was conducted, the estimated participation percentage should be indicated.

Reply: We appreciate the reviewer’s comment regarding the STROBE guidelines. We have reviewed the checklist for cross-sectional studies and ensured that our manuscript aligns with its recommendations. As noted in our response to the previous reviewer, we have added a statement in the revised manuscript to confirm that we followed the STROBE guidelines in writing our manuscript:

Changes in the manuscript: see p. 4: “This report complies with the Checklist for Reporting of Survey Studies [45] and follows the Strengthening the Reporting of Observational Studies in Epidemiology (STROBE) reporting guideline for cross-sectional studies.”

Added reference: see p. 22: 45. Von Elm E, Altman DG, Egger M, Pocock SJ, Gøtzsche PC, Vandenbroucke JP, et al. The Strengthening the Reporting of Observational Studies in Epidemiology (STROBE) Statement: Guidelines for Reporting Observational Studies. PLoS Med. 2007 Oct 16;4(10):e296).

Regarding the participant scenario, we adopted a convenience sampling rather than a census, which means that participants were recruited based on their availability and willingness to participate, rather than targeting a specific proportion of the broader population. As a result, we did not estimate participation rates per country, nor did we anticipate the exact number of respondents for each country. Although we did not perform analyses by country, we acknowledge the potential implications of this diversity, which we discuss in the discussion of the manuscript.

We have clarified the sampling methods in the manuscript:

Changes in the manuscript: see p. 6: “This study employed convenience sampling, and as such, no census was conducted. Consequently, we did not estimate the exact participation rate per country, nor did we track the number of potential participants by country. The study's design did not aim to perform country-specific analyses, as this was not within the scope of the research objectives. Nevertheless, we acknowledge that the inclusion of participants from different countries may have introduced variability related to differing healthcare systems, cultural norms, and medical practices, an issue that we discuss further in the discussion**.”**

We hope this clarifies the study design and provides sufficient transparency on the participant scenario and sampling approach.

While a sample size calculation is mentioned, insufficient detail is provided about the parameters used to determine the minimum sample size, which is critical for methodological transparency.

Reply: We agree that providing sufficient detail about the parameters used for determining the minimum sample size is critical for methodological transparency. As noted in the supplementary materials, the sample size calculation was based on established guidelines for Exploratory Factor Analysis (EFA) and correlational analyses. Full details can be found in the study protocol, which is available online.

The minimum sample size for our study was determined based on the need to conduct EFAs (as explained in the manuscript, at the time of the preregistration, CFAs were not planned) as well as correlational and regression analyses. Specifically, we aimed for a minimum sample size of 225 participants, based on the following calculations:

For the EFA (involving 35 items and 3 expected factors), the sample size required for good factor recovery was estimated at 175 participants, based on the guidelines of Child (2006) and Watkins (2021). Given the combination of multiple analyses to be conducted, we planned to recruit up to 50 additional participants beyond the minimum estimate, to account for potential exclusions and dropout. For the correlation analyses, calculation in the software program G*Power suggested that 211 participants would be required to achieve a power of 0.90 for detecting a medium effect size (r = 0.2). The sample size for the linear multiple regressions could not be precisely predicted before the EFA results, as we did not yet know how many factors would emerge from the EFAs to represent the professional norms of the medical culture. However, based on our expectations of having 3 independent factors (representing cultural dimensions), plus the control variable (social desirability), the required sample size for linear regression with 4 independent variables was estimated at 124 participants (using G*Power with an expected small to medium effect size of F² = 0.13). Given the need for sufficient statistical power across various analyses, we determined that a sample size of at least 225 participants would provide adequate power for all statistical procedures.

We hope this additional clarification helps to address the reviewer’s concern. To maintain the clarity of the manuscript and avoid overburdening the main text with technical details, we have decided to include a more comprehensive description of the sample size calculation in the supplementary materials:

Changes in the supplementary material: see p. 1: “The sample size for this study was determined based on the need to conduct Exploratory Factor Analyses (EFA), correlational analyses, and linear multiple regressions, following guidelines for each statistical procedure. For the EFA, which involved a total of 35 items and 3 expected factors, the sample size required for good factor recovery was estimated at 175 participants, based on the guidelines of Child (2006) and Watkins (2021). Since multiple analyses were to be conducted, we planned to recruit up to 50 additional participants beyond this minimum estimate to account for potential exclusions and dropout. For the correlational analyses, G*Power was used to determine the sample size required to achieve a power of 0.90 for detecting a medium effect size (r = 0.2). Based on this calculation, the minimum sample size required was 211 participants. The sample size for the linear multiple regressions could not be precisely predicted before the EFA results, as the number of factors representing the professional norms of the medical culture was not yet known. However, based on our expectations of having 3 independent factors (representing cultural dimensions) plus the control variable (social desirability), the sample size for linear regression with 4 independent variables was estimated at 124 participants, using G*Power with an expected small to medium effect size (F² = 0.13).Given the combination of these analyses, the minimum required sample size for the study was estimated to be 225 participants, and we aimed to recruit up to 550 participants to account for possible exclusions.”

c) The fit indices used in the confirmatory (CFA) and exploratory factor analyses (EFA) are important and should be included in the methods section rather than being relegated to supplementary material.

Reply: Thank you for this insightful comment. We agree that the fit indices used in the exploratory and confirmatory factor analyses (EFA and CFA) are essential for methodological transparency, and merit greater visibility in the main text. To address this, we incorporated a concise description of the fit indices and their thresholds directly into the methods section of the revised manuscript. Additionally, detailed results for all tested models (currently in the supplementary materials) will remain available for readers who wish to delve deeper into the statistical comparisons and justification for the retained model. This approach ensures that the manuscript remains accessible, while providing full transparency for close scrutiny by interested readers.

In the methods section, the following information was added regarding criteria for the EFA:

Changes in the manuscript: see p.7: “We assessed suitability of our data with the Kaiser-Meyer-Olkin (KMO) measure of sampling adequacy and Bartlett’s test of sphericity. Item loadings of ≥ 0.40 and communalities > 0.20 were required; cross-loading items were defined as those items loading ≥ 0.30 on two or more factors. Such items were considered unstable and were therefore removed. The proportion of total variance explained (TVE) by the factors needed to be ≥ 50%. To determine the number of factors to retain, we relied on various methods: Kaiser’s criterion, the scree plot, and the parallel analysis [50–53].”

And regarding criteria for the CFA:

Changes in the manuscript: see p.7: “We compared five statistical models (outlined in S1 through S5 figures and described in S5 table) using different goodness-of-fit indices to determine the statistical acceptability of the models and to retain the best-fitting model: the chi-square indices (χ2)[54], the root mean square error of approximation (RMSEA), the standardized root mean square residual (SRMR), the comparative fit index (CFI), and the Tucker-Lewis index (TLI) [55]. Acceptable fit criteria included RMSEA and SRMR ≤ 0.08, CFI and TLI > 0.90 [54,55].”

In the results section, we included more detailed information regarding the fitting indices of the final EFA solution:

Changes in the manuscript: see p. 10: “… The final structure comprised eight factors including 24 items. The Kaiser-Meyer-Olkin (KMO) measure of sampling adequacy of this structure was high (0.84), and Bartlett’s test of sphericity was highly significant, χ² (276) = 5069.83, p < 0.000. This solution accounted for 59% of item variance and yielded a simple structure (with no cross-loading items).”

d) The table 1 presents percentage data for explanatory variables for the total sample, but it also includes a specific subset (residents) and continuous variables, which makes it confusing to interpret. Suggested improvements include: Separating the general data from the subset (residents) with clear titles indicating the differentiation; Removing continuous variable values from the table, as the text already sufficiently presents medians and IQRs. Another advice is to make the table title more informative by detailing its content.

Reply: We thank the reviewer for these suggestions to improve the clarity and readability of Table 1. We have restructured the table by separating general sociodemographic and work-related characteristics for the total sample from the subsets of specialist physicians and of general practitioners (detailing the proportion of attending vs resident physicians in each subset), each section now having clear titles. We have also removed continuous variable values (medians and IQRs) from the table, as they are already described in the text. Finally, we have revised the table title to better reflect its detailed content:

Changes in the manuscript: see pp. 8-10:

Table 1. Sociodemographic and work-related characteristics of the 1002 surveyed physicians, including detailed subsets for specialist physicians and general practitioners.

| **Sociodemographic and work-related characteristics (total sample, N = 1002)** | **N (%)** |
| --- | --- |
| **Gender** | |
| Male | 347 (34.6) |
| Female | 547 (64.6) |
| Other | 2 (0.2) |
| Missing | 6 (0.6) |
| **Relationship status** | |
| Single | 175 (17.5) |
| In a relationship | 825 (82.3) |
| Missing | 2 (0.2) |
| **Parental status** | |
| Yes | 582 (41.4) |
| No | 415 (58.1) |
| Missing | 5 (0.5) |
| **Country of residence** | |
| Belgium | 922 (92) |
| France | 65 (6.5) |
| Switzerland | 2 (0.2) |
| Other | 6 (0.6) |
| Missing | 7 (0.7) |
| **Type of physician** | |
| General practitioner | 339 (33.8) |
| Specialist physician | 646 (64.5) |
| Occupational medicine physicians / Insurance physicians / School physicians / Medical consultants | 14 (1.4) |
| Missing | 3 (0.3) |
| **Work-related characteristics (specialist physicians only, N = 646)** | **N (%)** |
| **Status** | |
| Attending | 458 (70.9) |
| Resident | 188 (29.1) |
| **Work setting** | |
| Hospital setting | 560 (86.6) |
| Private consultation | 13 (2.0) |
| Both | 60 (9.3) |
| Missing | 13 (2.0) |
| **Type of hospital** | |
| Public hospital | 204 (32.9) |
| Private hospital | 127 (20.5) |
| University hospital | 288 (46.5) |
| Missing | 27 (4.2) |
| **Specialty** | |
| Anesthesiology | 60 (9.3) |
| Pediatrics | 52 (8.1) |
| Surgery | 49 (7.6) |
| Acute and emergency medicine | 45 (7.0) |
| Neurology and pediatric neurology | 40 (6.2) |
| Psychiatry | 37 (5.7) |
| Internal medicine | 35 (5.4) |
| Gynecology | 25 (3.9) |
| Intensive care | 24 (3.7) |
| Cardiology | 19 (2.9) |
| Gastro-enterology | 19 (2.9) |
| Geriatrics | 18 (2.8) |
| Otolaryngology | 18 (2.8) |
| Pneumology | 18 (2.8) |
| Hematology | 16 (2.5) |
| Physical medicine and rehabilitation | 15 (2.3) |
| Dermatology | 13 (2.0) |
| Endocrinology | 13 (2.0) |
| Radiology | 13 (2.0) |
| Clinical biology | 11 (1.7) |
| Clinical infectiology | 11 (1.7) |
| Oncology | 11 (1.7) |
| Ophthalmology | 10 (1.6) |
| Urology | 9 (1.4) |
| Nuclear medicine | 8 (1.2) |
| Nephrology | 8 (1.2) |
| Pathological anatomy | 7 (1.1) |
| Neurosurgery | 6 (0.9) |
| Radiotherapy – oncology | 5 (0.8) |
| Rheumatology | 3 (0.5) |
| Neonatology | 2 (0.3) |
| Stomatology | 2 (0.3) |
| Palliative care | 2 (0.3) |
| Other (hospital hygiene, medical information, orthodontics, legal medicine) | 4 (0.6) |
| Missing | 18 (2.8) |
| **Work-related characteristics (general practitioners only, N = 339)** | **N (%)** |
| **Status** | |
| Attending | 278 (82.0) |
| Resident | 61 (18.0) |
| **Type of practice**^a^ | |
| General medicine (consultations and home visits) | 327 (96.5) |
| Hospital setting | 14 (4.1) |
| Other institution (e.g. family planning center, nursing home) | 52 (15.3) |
| Non-conventional medicine | 11 (3.2) |
| **Work setting**^a^ | |
| Works alone | 90 (26.5) |
| Monodisciplinary group | 107 (31.6) |
| Multidisciplinary group | 150 (44.2) |

**Legend Table 1.** N of the total sample = 1002, N of the subset of specialist physicians = 646, N of the subset of general practitioners = 993. ^a^Multiple responses were possible.

e) A particularly relevant issue that requires further explanation is the correlation of the second-order dimension C with the global HDMC score, which is considerably lower than the other dimensions. The authors

should discuss possible reasons for this discrepancy, considering both theoretical and methodological factors.

Reply: Thank you for your comment regarding the lower correlation of dimension C (Physician Stigma towards Burnout) with the global HDMC score. We appreciate your insight, and have carefully considered both methodological and theoretical explanations for this discrepancy.

**Methodological considerations:** Physician Stigma towards Burnout had a lower internal consistency (α = 0.62) compared to dimensions A (Professional Commitment; α = 0.85) and B (Myth of the Invulnerable Physician; α = 0.77). This lower reliability, which could weaken its correlation with the global HDMC score, aligns with previous studies on stigmatizing attitudes among physicians, which have also noted challenges in reliably measuring these phenomena in physicians (e.g. Clough et al., 2020; Favre et al., 2023). Additionally, dimension C comprises only four items, whereas dimensions A and B include 11 and 6 items, respectively. This smaller number of items may make the measurement of dimension C less stable, further influencing its correlation.

**Theoretical considerations:** Physician Stigma towards Burnout focuses narrowly on stigmatizing attitudes specific to burnout, which was our theoretical choice at an early stage of the study design, in consideration of the literature and perspectives of physicians. This dimension may be less central to the broader harmful aspects of medical culture than dimensions A and B. In other words, burnout stigma may not be as central within medical culture as the norms of professional commitment and invulnerability, resulting in a weaker association with the global HDMC score.

We have added a paragraph in the results section to highlight the observed discrepancy:

Changes in the manuscript: see pp. 11-12: “Strong positive correlations were found between the global HDMC score and the dimensions of Professional Commitment (*ρ* = 0.84, p< 0.001), and the Myth of the Invulnerable Physician (*ρ* = 0.75, p< 0.001), respectively, suggesting their central role in the concept of harmful medical culture. In contrast, Physician Stigma towards Burnout showed a significant but notably lower correlation (*ρ* = 0.56, p< 0.001) suggesting that, while still important, it represents a less central dimension compared to the others two.”

We have also included these methodological and theoretical explanations in the discussion section to contextualize the findings:

Changes in the manuscript: see pp. 15-16: “We found strong positive correlations between the global HDMC score and the dimensions of Professional Commitment and the Myth of the Invulnerable Physician, underscoring their centrality to the concept of harmful medical culture. In contrast, Physician Stigma towards Burnout showed a weaker correlation. This may partly stem from methodological factors, such as the smaller number of items and its lower internal consistency, which may have contributed to greater variability and weaker correlations. Theoretically, stigma towards burnout may be less central to harmful medical culture than the broader constructs of professional commitment and invulnerability, which are historically deeply ingrained in the profession.”
